# Genomic and proteomic analysis of lignin degrading and polyhydroxyalkanoate accumulating β-proteobacterium *Pandoraea* sp. ISTKB

**DOI:** 10.1186/s13068-018-1148-2

**Published:** 2018-06-05

**Authors:** Madan Kumar, Sandhya Verma, Rajesh Kumar Gazara, Manish Kumar, Ashok Pandey, Praveen Kumar Verma, Indu Shekhar Thakur

**Affiliations:** 1 School of Environmental Sciences, Jawaharlal Nehru University, New Delhi, 110067, India; 2 National Institute of Plant Genome Research, Aruna Asaf Ali Marg, New Delhi, 110067 India; 3 CSIR-Indian Institute of Toxicology Research, 31 MG Marg, Lucknow, 226 001 India

**Keywords:** Genomics, Lignin, Polyhydroxyalkanoate, Gene cluster, Label-free quantification, Vanillic acid

## Abstract

**Background:**

Lignin is a major component of plant biomass and is recalcitrant to degradation due to its complex and heterogeneous aromatic structure. The biomass-based research mainly focuses on polysaccharides component of biomass and lignin is discarded as waste with very limited usage. The sustainability and success of plant polysaccharide-based biorefinery can be possible if lignin is utilized in improved ways and with minimal waste generation. Discovering new microbial strains and understanding their enzyme system for lignin degradation are necessary for its conversion into fuel and chemicals. The *Pandoraea* sp. ISTKB was previously characterized for lignin degradation and successfully applied for pretreatment of sugarcane bagasse and polyhydroxyalkanoate (PHA) production. In this study, genomic analysis and proteomics on aromatic polymer kraft lignin and vanillic acid are performed to find the important enzymes for polymer utilization.

**Results:**

Genomic analysis of *Pandoraea* sp. ISTKB revealed the presence of strong lignin degradation machinery and identified various candidate genes responsible for lignin degradation and PHA production. We also applied label-free quantitative proteomic approach to identify the expression profile on monoaromatic compound vanillic acid (VA) and polyaromatic kraft lignin (KL). Genomic and proteomic analysis simultaneously discovered Dyp-type peroxidase, peroxidases, glycolate oxidase, aldehyde oxidase, GMC oxidoreductase, laccases, quinone oxidoreductase, dioxygenases, monooxygenases, glutathione-dependent etherases, dehydrogenases, reductases, and methyltransferases and various other recently reported enzyme systems such as superoxide dismutases or catalase–peroxidase for lignin degradation. A strong stress response and detoxification mechanism was discovered. The two important gene clusters for lignin degradation and three PHA polymerase spanning gene clusters were identified and all the clusters were functionally active on KL–VA.

**Conclusions:**

The unusual aerobic ‘-CoA’-mediated degradation pathway of phenylacetate and benzoate (reported only in 16 and 4–5% of total sequenced bacterial genomes), peroxidase-accessory enzyme system, and fenton chemistry based are the major pathways observed for lignin degradation. Both *ortho* and *meta* ring cleavage pathways for aromatic compound degradation were observed in expression profile. Genomic and proteomic approaches provided validation to this strain’s robust machinery for the metabolism of recalcitrant compounds and PHA production and provide an opportunity to target important enzymes for lignin valorization in future.

**Electronic supplementary material:**

The online version of this article (10.1186/s13068-018-1148-2) contains supplementary material, which is available to authorized users.

## Background

The genus *Pandoraea* is a very recently classified genus proposed in the year 2000. Bacteria belonging to genus *Pandoraea* are Gram-negative, non-sporulating, and motile bacteria with single polar flagellum [1]. The genus belongs to *Burkholderiaceae* family and class β-proteobacteria. The *Pandoraea* genus was earlier misidentified and grouped together with *Burkholderia* or *Ralstonia* [1] This genus contains five species (*Pandoraea pnomenusa*, *Pandoraea sputorum*, *Pandoraea norimbergensis*, *Pandoraea apista,* and *Pandoraea pulmonicola*) and four genomospecies of thiosulfate-oxidizing (*Pandoraea thiooxydans*) and oxalate-oxidizing species as *Pandoraea vervacti*, *Pandoraea faecigallinarum, and Pandoraea oxalativorans*. *Pandoraea* is a taxonomically distinct genus having close similarity with *Burkholderia* and *Ralstonia*. *Pandoraea* has been isolated from various environments such as soil, landfill site, sediments, clinical samples (only *P. apista*, *P. pnomenusa,* and *P. sputorum* isolated until date), and water [1–4]. The *Burkholderia* and *Ralstonia* are very much explored and established genera with their promising potential environmental and industrial applications. *Pandoraea* is a relatively new genus, so there are very few findings available about their biotechnological potential. The species from this genus have been documented for utilization of polychlorinated biphenyl, dichloromethane, dyes, lignin, oxalate, thiosulfate, and quorum sensing [3–6]. At present, the genomic insights for *Pandoraea* are limiting and such studies would eventually help to widen the biotechnological prospective of this genus.

Lignin is a complex aromatic heteropolymer and it is the most abundant aromatic polymer available on earth. In nature, lignin is degraded mainly by bacteria and fungi. Fungi have been studied extensively for lignin degradation and only a few bacterial species have been reported for lignin degradation [7, 8]. Compared to fungi, bacteria offer advantage as its genome size is small, genetic manipulations, and large-scale recombinant expression of important enzymes can be performed with a greater ease. Therefore, the focus again shifted to bacteria for the identification of novel strains and enzymes for lignin degradation. The discovery of novel ligninolytic microbes, enzymes, and their biochemical characterization will help in deconstruction of biomass for their application in biofuel and bioproduct industry [6, 9–11]. The application of advanced ‘omics’ approach such as genomics, transcriptomics, and proteomics to individual microbial strains or microbial community will help in identification and functional characterization of novel ligninolytic enzymes in the near future [12–14]. With the increase in genomic data of bacteria and fungi, the biomass degrading potential across different taxa can be identified that will further enhance our understanding related to lignin degradation [12, 13]. The lignin degrading bacterial isolate belongs to actinobacteria, alpha proteobacteria, beta proteobacteria, gamma proteobacteria, delta proteobacteria, bacteroides, and archaea [7]. The novel bacterial enzymes responsible for lignin degradation and their mechanism of action have also been described [15]. In recent years, LC–MS-based proteomics studies have been widely performed. Quantitative LC–MS-based proteomics such as label free and ITRAQ labeling-based quantification methods are generally used to identify the novel enzymes and their level of expression in a particular process [16–18].

We have earlier sequenced the genome of *Pandoraea* sp. ISTKB and the sequence has been submitted to NCBI with accession number MAOS00000000.1 which is openly available [19]. In the present study, we describe the comprehensive analysis of the *Pandoraea* sp. ISTKB genome. The bioinformatics analysis was performed to identify a large set of genes and pathways putatively responsible for lignin degradation and PHA production. The important gene clusters responsible for lignin degradation and PHA production were also highlighted. This strain has already been shown to utilize monoaromatic lignin derivatives with great ease compared to polymeric kraft lignin for PHA production [20]. Therefore, the proteomic study of *Pandoraea* sp. ISTKB was performed for identification of set of a proteins expressed during its growth on monoaromatic vanillic acid (VA) and aromatic polymer lignin, i.e., kraft lignin (KL) that can be overexpressed for enhanced KL utilization. VA was selected, because most of the lignin linkages proceed through generation of vanillin or VA as nodal point during the course of degradation [21]. Proteomic studies provide insight into the protein profile and also complement the genomics analysis. Genomic and proteomic analyses would enable us to understand the novel enzymes and pathways responsible for lignin degradation and biovalorization.

## Results

### Salient features of *Pandoraea* sp. ISTKB genome

**Fig. 1:**
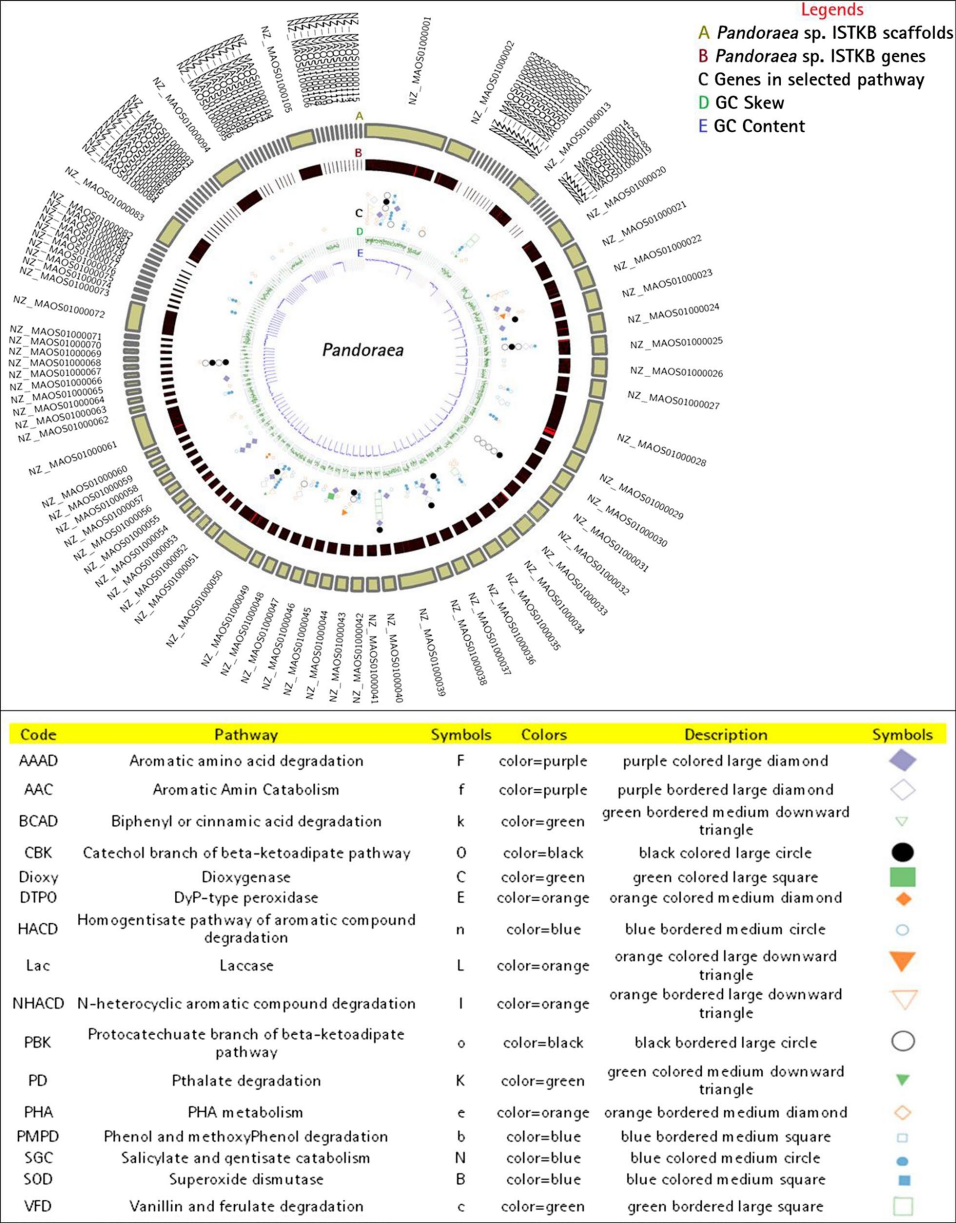
Circos plot of genes compared with the genome for *Pandoraea* sp. ISTKB. Circles from outside to inside represent; **a** scaffold arrangement, **b** gene position on the scaffolds, **c** GC skew, and **d** GC content. Syntenic representation of genes associated with the pathways and *Pandoraea* sp. ISTKB. Different genes associated with the selected pathways with different colors and shapes

**Fig. 2:**
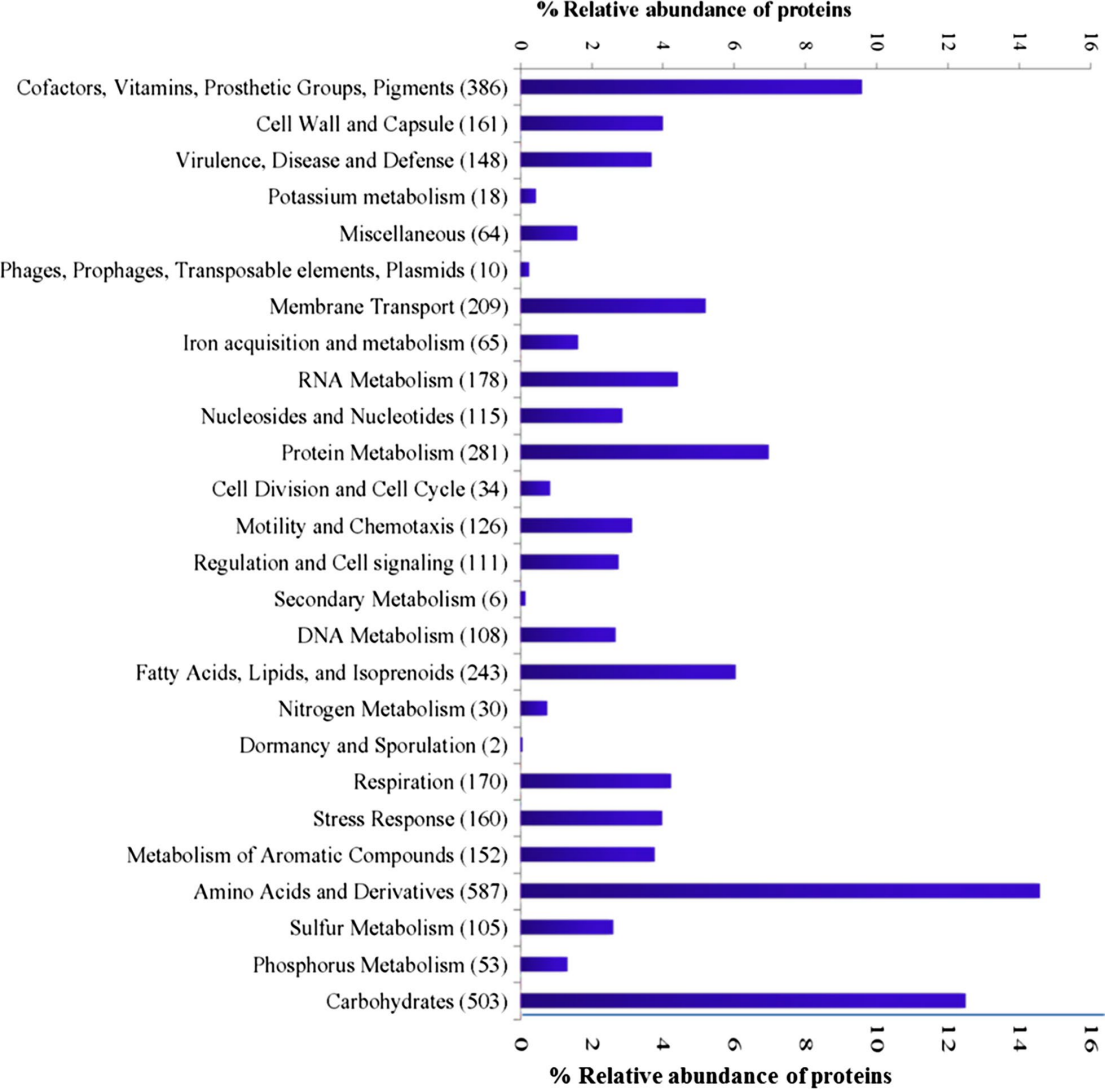
Classification of proteins in subsystem features and their abundance in different functional groups shown in *Pandoraea* sp. ISTKB

The *Pandoraea* sp. ISTKB was previously characterized for lignin degradation and successfully applied for pretreatment of sugarcane bagasse and polyhydroxyalkanoate (PHA) production [6, 20, 22]. The genome size of *Pandoraea* sp. ISTKB is 6.37 Mb with 65× coverage having GC content of 62.05%, 5356 predicted protein-coding genes [prokaryotic genome annotation pipeline (PGAP) and Pfam annotation] and the other general genome features has also been reported earlier [19]. Among the predicted proteins, 1740 proteins were categorized as hypothetical proteins. Out of total predicted proteins, 456 proteins were identified having signal sequences. Circular map displaying genomic features provides a space efficient and clear representation of gene arrangement on the genome, as shown in Fig. 1. The annotation of important genes and pathways related to lignin or aromatic compound degradation has also been represented in the circular plot. KEGG–KAAS pathway analysis of protein-coding genes from *Pandoraea* sp. ISTKB categorized 2590 genes in 22 different functional KAAS pathway (Additional file 1: Table S1). The KEGG predicted 148 proteins responsible for degradation and metabolism of aromatic and xenobiotic compound. The annotation and analysis by RAST predicted 5658 coding genes and 48% of coding genes have been classified into 26 subsystems features. The percent contribution of genes present in different functional groups in subsystem features is represented in Fig. 2. The subsystem features count showed dominance of general process related to carbohydrate, amino acids, cell wall components, prosthetics, cofactors, proteins, and lipid metabolism. After normal cellular processes, the subsystem feature count is dominated by membrane transport, aromatic compound metabolism, respiration stress response regulation, and cell signaling.

**Fig. 3:**
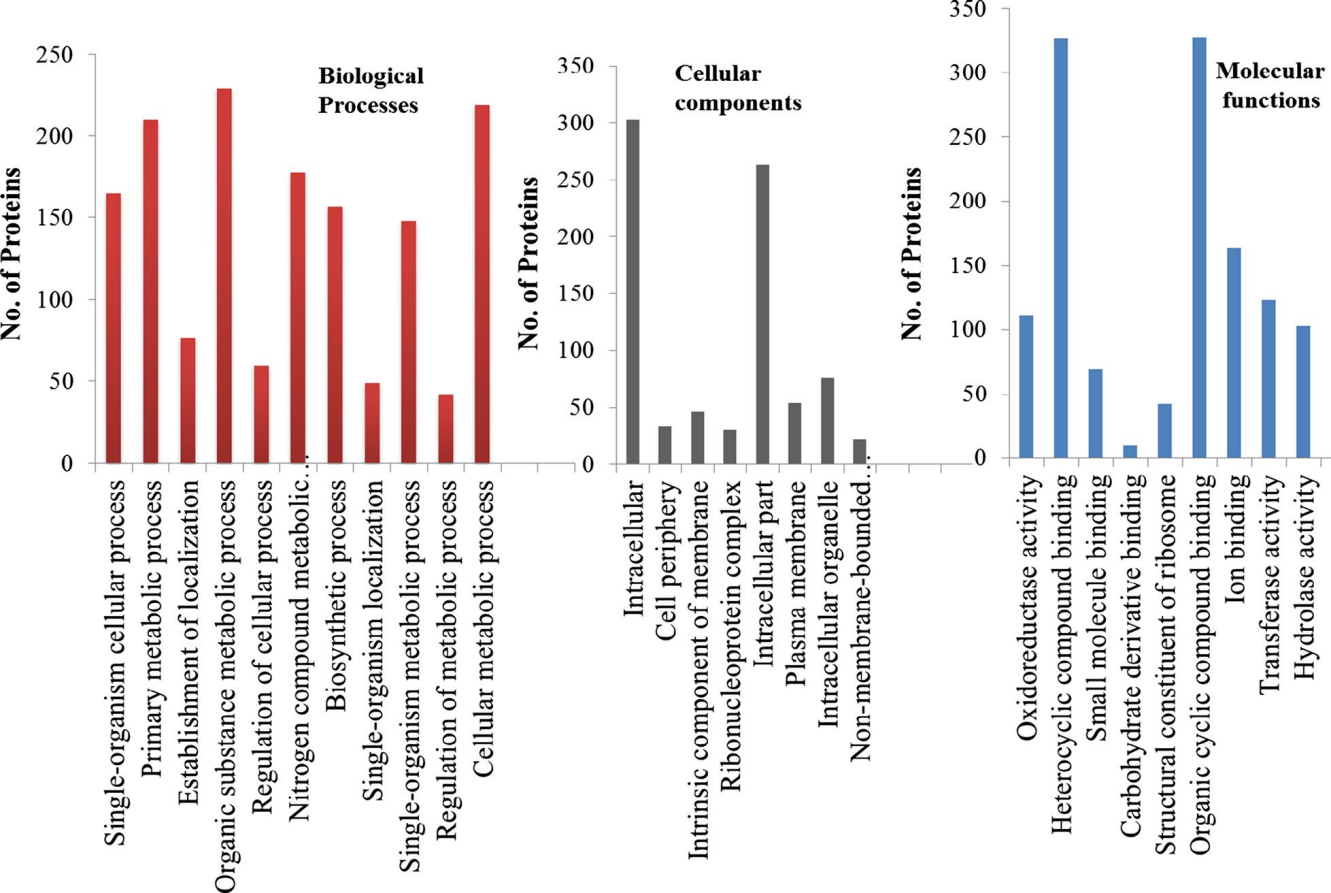
GO analysis of *Pandoraea* sp. ISTKB genome and classification of genes into biological processes, cellular components, and molecular functions

Gene ontology (GO) analysis was performed to gain functional information about predicted proteins in the genome. The analysis provided information about distribution of genes among various metabolic processes, cellular functions, and molecular components in the genome of *Pandoraea* sp. ISTKB (Fig. 3). In the biological processes, the organic substance metabolic process was found to be the dominant process. Molecular functions analysis revealed the major distribution of proteins into three important functions, i.e., organic cyclic compound binding, heterocyclic compound binding, and oxidoreductase activity. Abundance of ion binding and small molecule-binding proteins indicates their role in transcriptional regulation and transportation of molecules across cell membrane. Representation of transferase and hydrolase in good proportion indicates their assistance during metabolism of organic compounds.

### Metabolism, respiratory mechanism, transporters, and transcriptional factors in *Pandoraea* sp. ISTKB genome

*Pandoraea* sp. ISTKB can metabolize diverse substrates; which includes five and six carbon sugar molecules. This bacterium can utilize monosaccharide (galactose, mannose, and fructose), disaccharides (sucrose), polysaccharides (starch), glucuronate, ascorbate, aldarate, amino sugar and nucleotide sugar, propionate, and butanoate metabolism. This strain can also utilize pentoses (xylose, xylulose), C5-branched dibasic acid, and other glyoxylate, dicarboxylate and pyruvate as predicted by KEGG. The growth of this strain was observed to be poor on glucose and the KEGG pathway analysis of carbohydrate metabolism also supported this observation. Analysis of respiratory mechanism showed various terminal electron acceptor, electron donors, and also other relevant genes related to respiration. The abundance of formate dehydrogenase, quinone oxidoreductase family proteins, oxidoreductases, ubiquinol oxidase, soluble cytochrome, and other related electron carriers highlights their importance and assistance in metabolism of various recalcitrant compounds (Additional file 1: Figure S1). There were 346 transcriptional factors identified in the genome, and among these regulators, LysR family was found to be dominant. Transcriptional regulator families related to metabolism of aromatic compound such as GntR, MarR, IclR, XRE, aromatic hydrocarbon utilization, anaerobic benzoate metabolism, and organic hydroperoxide regulators are also present in this strain (Additional file 1: Figure S2). There are 587 transporters identified in the genome, and among these, there were 279 ABC family transporters present. This family represents almost half of the total transporters present in the genome and was found to be dominant followed by two-component system and MFS transporters (Additional file 1: Figure S3).

### Metabolism of aromatic compounds

The annotation of *Pandoraea* sp. ISTKB genes and their classification into pathways involved in lignin or aromatic compounds degradation have been identified by KEGG pathway analysis, blast search against ‘nr’ database, and subsystem feature of RAST. There were 42 dioxygenase, 25 monooxygenase, 17 peroxidase (including one DyP-type peroxidase), and 2 laccases discovered in genome (Additional file 1: Figure S4; Tables S2, S3, and S4). The presence of various oxidoreductase [grouped into FAD, NAD(P)H, SDR, GMC, YggW, quinone, pyridine nucleotide–disulfide, flavin, Fe–S, and unclassified oxidoreductases), reductases, dedydrogenases, esterases, thioesterases, transferases, and hydrolases has also been observed.

**Fig. 4:**
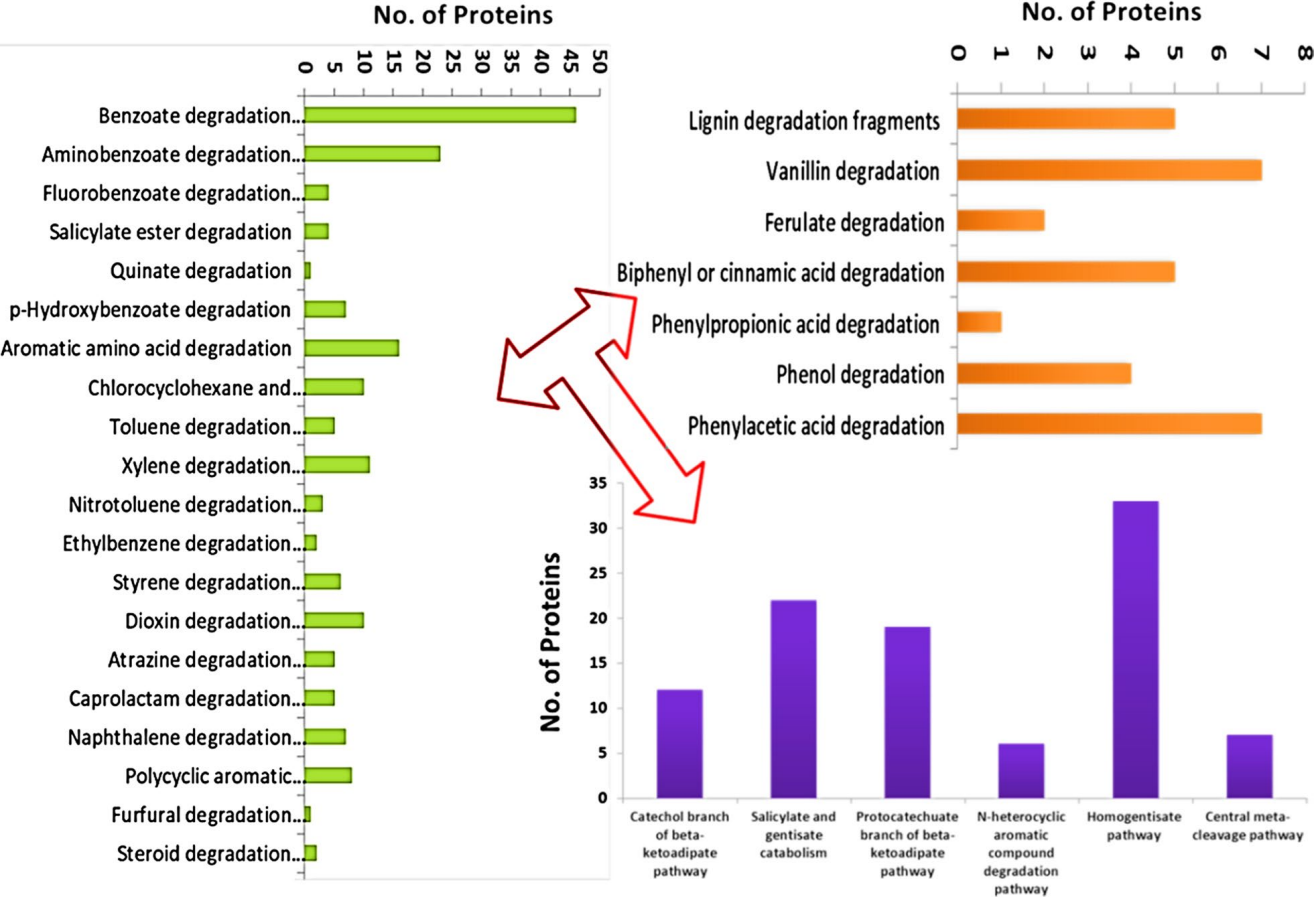
Predicted lignin and aromatic compounds degradation genes and their number responsible for funneling into peripheral pathways and central intermediate metabolism

The pathway analysis revealed genes responsible for lignin degradation and diverse aromatic compound metabolism (Fig. 4). Genes responsible for funneling of lignin or aromatic components’ degradation through peripheral degradation pathways have been observed. Genes related to pathways for degradation of vanillin, ferulate, biphenyl, phenylpropanoic acid, benzoyl-CoA mediated, phenylacetate, and phenol were observed and their abundance is depicted in Fig. 4 and Additional file 1: Table S5. Subsystem feature analysis identified genes as ‘lignin degradation fragments’ responsible for lignin metabolism and this is discussed as cluster later section. The KEGG analysis indicates that this strain can utilize various xenobiotic compounds such as benzoate derivatives (amino, ethyl, *p*-hydroxy, and fluoro), BTX, salicylate esters, quinate, pesticides, PAHs, synthetic aromatic monomer, furfural, and steroids. The degradation of lignin and xenobiotic aromatic compounds results into generation of some restricted common central intermediates (catechol, protocatechuate, and gentisate) that are further metabolized by beta-ketoadipate and aromatic ring cleaving pathways. The genes responsible for degradation of central intermediates were identified in abundance (Fig. 4 and Additional file 1: Table S6). The genes observed in central intermediate pathways can metabolize common aromatic intermediates through both *ortho* and *meta* cleavage pathways [23]. The genes responsible for metabolism of central intermediates such as catechol, protocatechuate, salicylate, homogentisate, *N*-heterocyclic aromatic compound, and *meta* cleavage pathways were also identified.

### Identification of stress response genes, secondary metabolites, and genomic islands

Lignin or aromatic compound degradation requires concerted action of various oxidoreductases. The degradation process generates free radicals and reactive intermediates and their removal or transformation into stable and less toxic component is essential for cell survival. Genome analysis identified various proteins related to stress response and detoxification mechanisms (Additional file 1: Figure S5 and Table S7). The presence of superoxide dismutase, catalases, glutathione, thioredoxin, peroxiredoxins, glyoxylases, rubrerythrin, glutaredoxins, aldo/keto reductase, and alkyl hydroperoxidase highlights this strain’s arsenal against oxidative stress, protection from reactive species and detoxification of toxic components during aromatic metabolism [24, 25].

There are nine gene clusters identified in the genome of *Pandoraea* sp. ISTKB that has been represented with their contigs and position marked in Additional file 1: Table S8. Secondary metabolite cluster analysis identified some novel metabolites that are specific to *Pandoraea* sp. ISTKB. These clusters included genes responsible for the synthesis of terpenes, nonribosomal peptides, thailanstatin/mangotoxin, arylpropane, 2 homoserine lactone, phosphonate–terpene, bacteriocin, and lassopeptide. The cluster 9 (lassopeptide), cluster 2 (Nrps), and cluster 4 (arylpropane) were found to be unique to this strain, since cluster 9 did not show any match with *Pandoraea* genus or *Burkholderia* genus. However, clusters 2 and 4 showed only one match with *Burkholderia*. Clusters 1 (terpenes), 3 (thailanstatin/mangotoxin), and 5 (homoserine lactone) are distributed among *Pandoraea* and *Burkholderia* genus. Moreover, clusters 6 (phosphonate–terpene), 7 (bacteriocin), and 8 (homoserine lactone) are highly represented in *Pandoraea* genus. The novel clusters such as cluster 9 (lassopeptide), 2 (Nrps), and 4 (arylpropane) can prove to be significant as these are unique to this strain.

There were 12 genomic islands identified in the genome that are mainly dominated by the hypothetical proteins (Additional file 1: Figure S6 and Additional file 2: Table S9). The other proteins present were related to DNA replication, cell division and partitioning, transposition, recombination, phage-mediated integration, repair, and DNA-binding response regulators. There are various proteins identified in the island that plays important role in stress response, detoxification mechanism and their regulation, electron carrier, antibiotic resistance, metal resistance, and transportation of molecules across cell membrane. The proteins related to phosphate and sulfur metabolism and few for aromatic compound degradation were also observed.

### Identification of gene clusters for the degradation lignin derivatives and PHA production

**Fig. 5:**
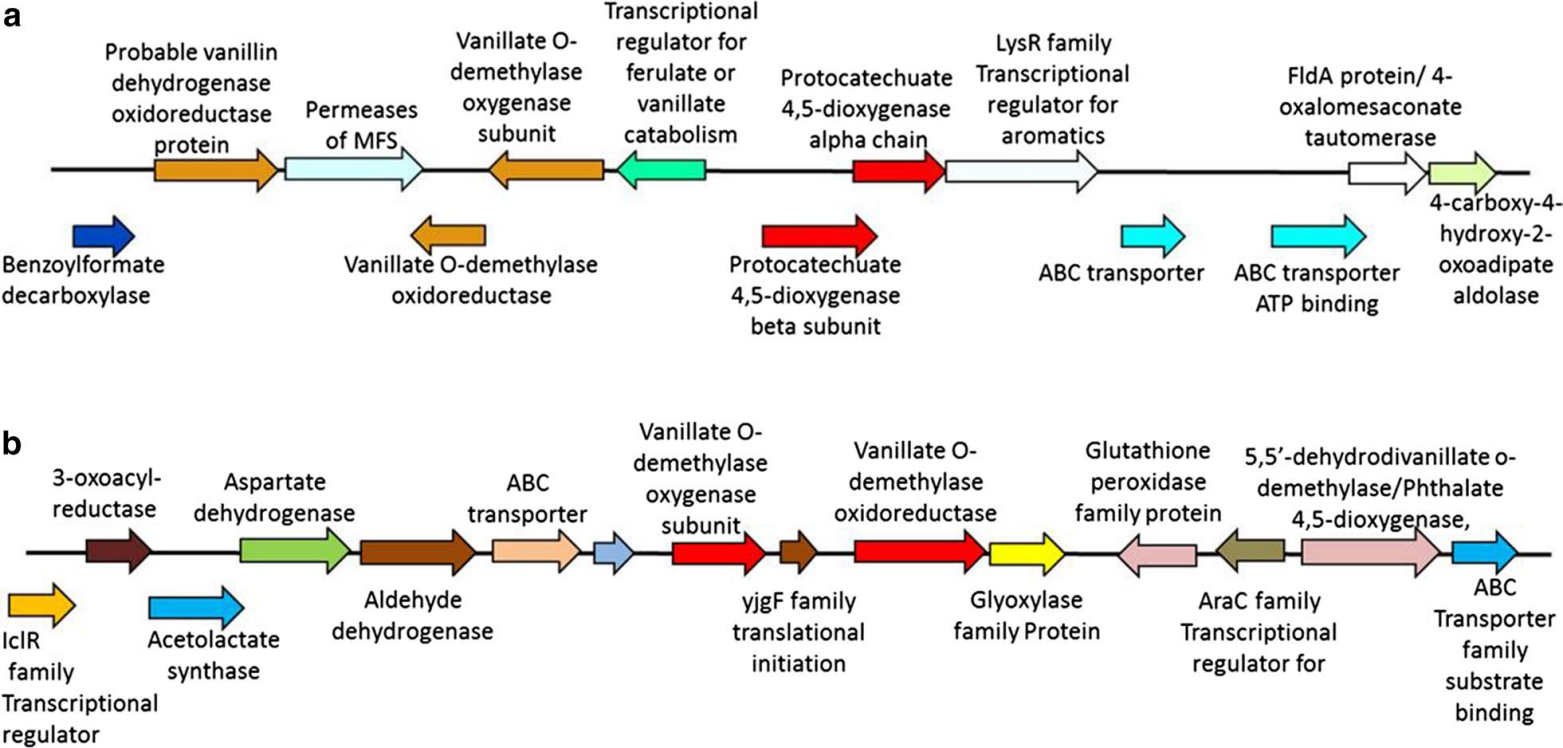
Gene clusters with contig number 40.1 and 13.1 identified in *Pandoraea* genome responsible for lignin degradation represented as **a** and **b**. The size of DNA fragment selected for cluster analysis is between 12 and 17 Kb

The two gene clusters responsible for degradation of lignin derivatives have been identified and the order of gene arrangement on the cluster is shown in Fig. 5a, b. The first cluster ‘lignin degradation fragment’ predicted by RAST contains genes responsible for protocatechuate *meta* cleavage-mediated degradation of lignin derivatives. The presence of LysR family transcriptional regulator for aromatics can be observed in the cluster. ABC transporters and MFS transporter were also present in this cluster that might be regulating the movement of aromatic compounds across the cell. The benzoyl formate decarboxylase present in the cluster is known for the degradation of benzene, xylene, and toluene. The second cluster contains genes mainly responsible for the degradation of vanillic acid. The presence of ABC transporters for regulating movement of molecules can also be observed in this cluster. This cluster also contains glutathione peroxidase, dehydrogenases, and glyoxylase that play important role in protection from oxidative damage by detoxifying reactive intermediates such as methylglyoxal and other aldehydes formed during metabolism of aromatic compounds [25].

**Fig. 6:**
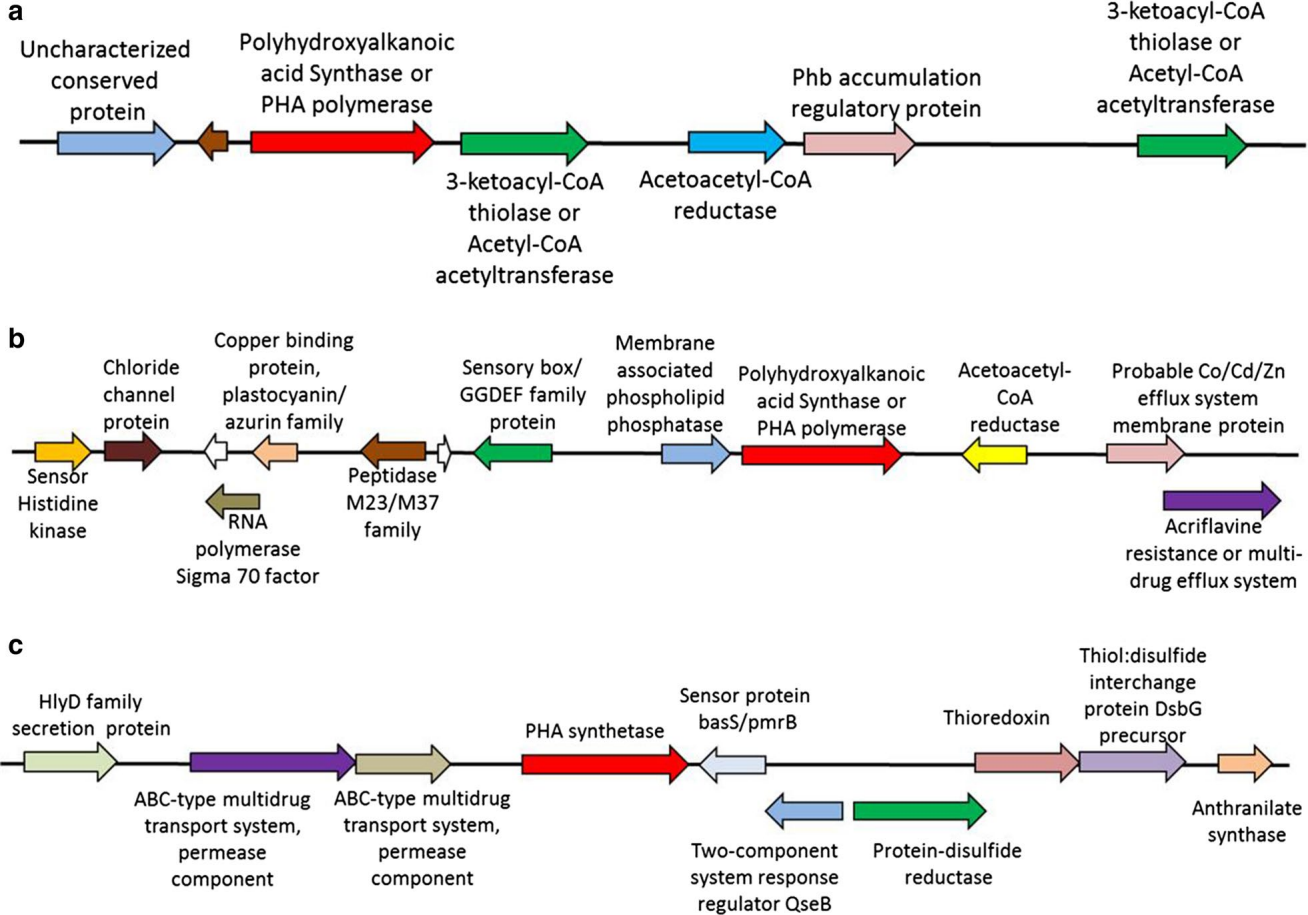
Gene clusters with contig numbers 23.1, 34.1, and 48.1 identified in *Pandoraea* genome responsible for PHA production represented as **a**–**c**. The size of DNA fragment selected for cluster analysis is between 12 and 17 Kb

PHA is carbon and energy reserve accumulated by microbes under nutrient imbalance condition [26]. We have earlier characterized PHA production by strain ISTKB while growing on lignin and its derivatives (as sole carbon source) and the genes responsible for PHA synthesis have been identified in the genome [20]. Here, the arrangement of PHA biosynthetic genes on cluster was analyzed in detail (Fig. 6a–c). The clusters were identified spanning PHA synthase or polymerase gene that is annotated in the genome. The first cluster revealed the presence of complete set of genes (acetoacetyl-CoA reductase, β-ketothiolase, PHA polymerase, and regulatory protein) responsible for short-chain PHA production. In case of second cluster, PHA polymerase was followed by acetoacetyl-CoA reductase but β-ketothiolase was missing from this cluster. The β-ketothiolase was present in multiple copies in the genome. This cluster is dominated by stress responsive proteins primarily related to heavy metal or multidrug efflux system. The third cluster contains only PHA synthetase and presence of genes predominantly related to oxidative stress as thiol-disulfide interchange protein, protein disulfide reductase, thioredoxin, two-component system response regulator protein, sensory proteins, secretory proteins, and ABC-type multidrug permeases was present around polymerase in the cluster.

### Proteomics analysis on kraft lignin and vanillic acid

**Fig. 7:**
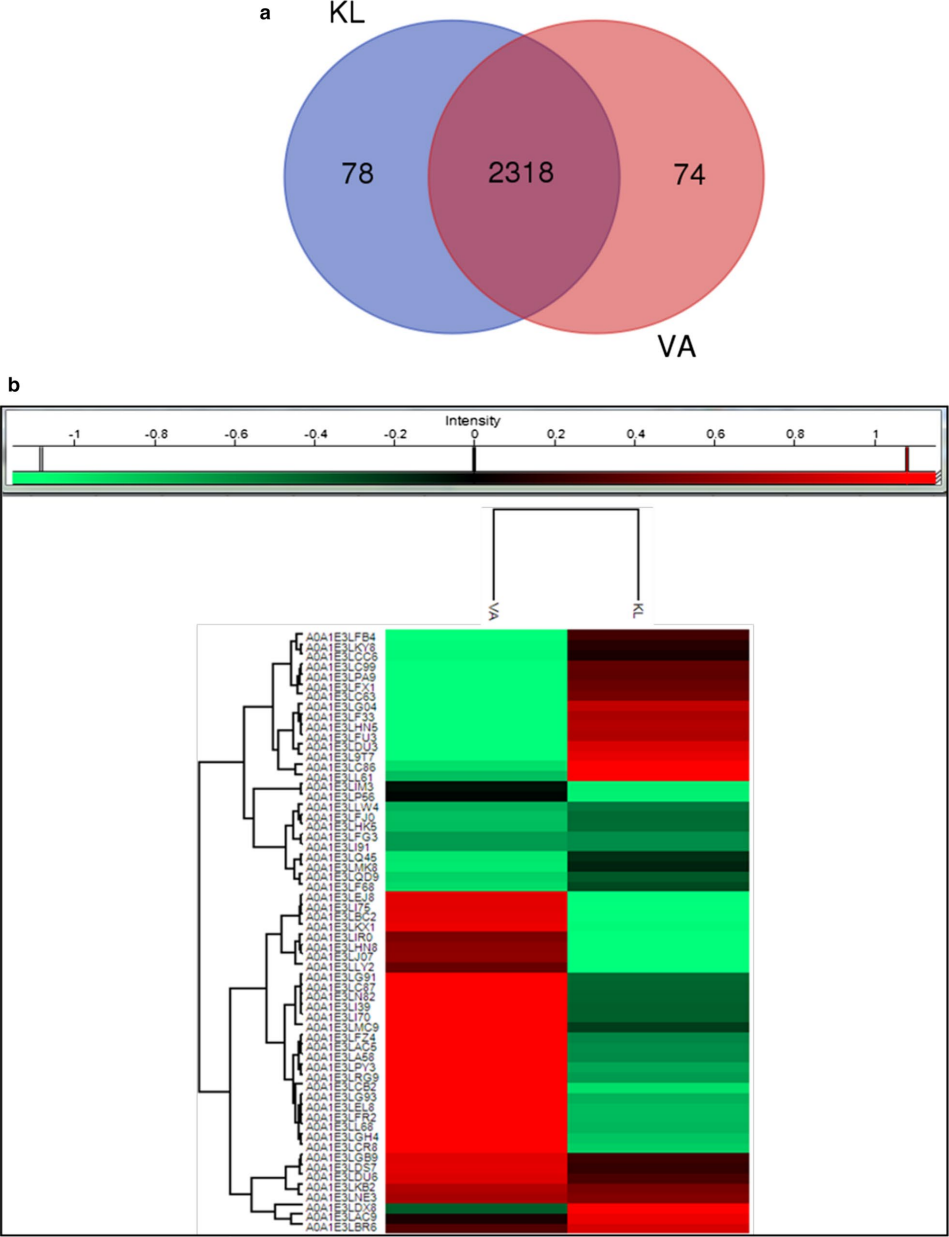
**a** Venn diagram showing total number of proteins expressed on kraft lignin and vanillic acid and their distribution among KL and VA. **b** Heat map showing differential expression of relevant proteins on kraft lignin–vanillic acid that are responsible for lignin degradation

Proteomic analysis was performed to identify the genes expressed on monoaromatic compound vanillic acid and polyaromatic compound kraft lignin. The identification of important proteins responsible for polymeric lignin degradation and their overexpression will provide opportunity for lignin valorization. There were total 2484 proteins detected during LC–MS analysis covering almost 44.61% of the total protein-coding genes present in the genome. There were 2318 proteins common in both KL and VA and 166 proteins were found to be expressed either on KL or on VA. Among 166 expressed proteins, 74 were expressed on VA and 78 proteins on KL, as shown in Fig. 7a, b. GO analysis was performed on the protein expressed on KL and VA to obtain the overview of functional information about the proteins involved in various biological processes, cellular components, and molecular functions.

**Fig. 8:**
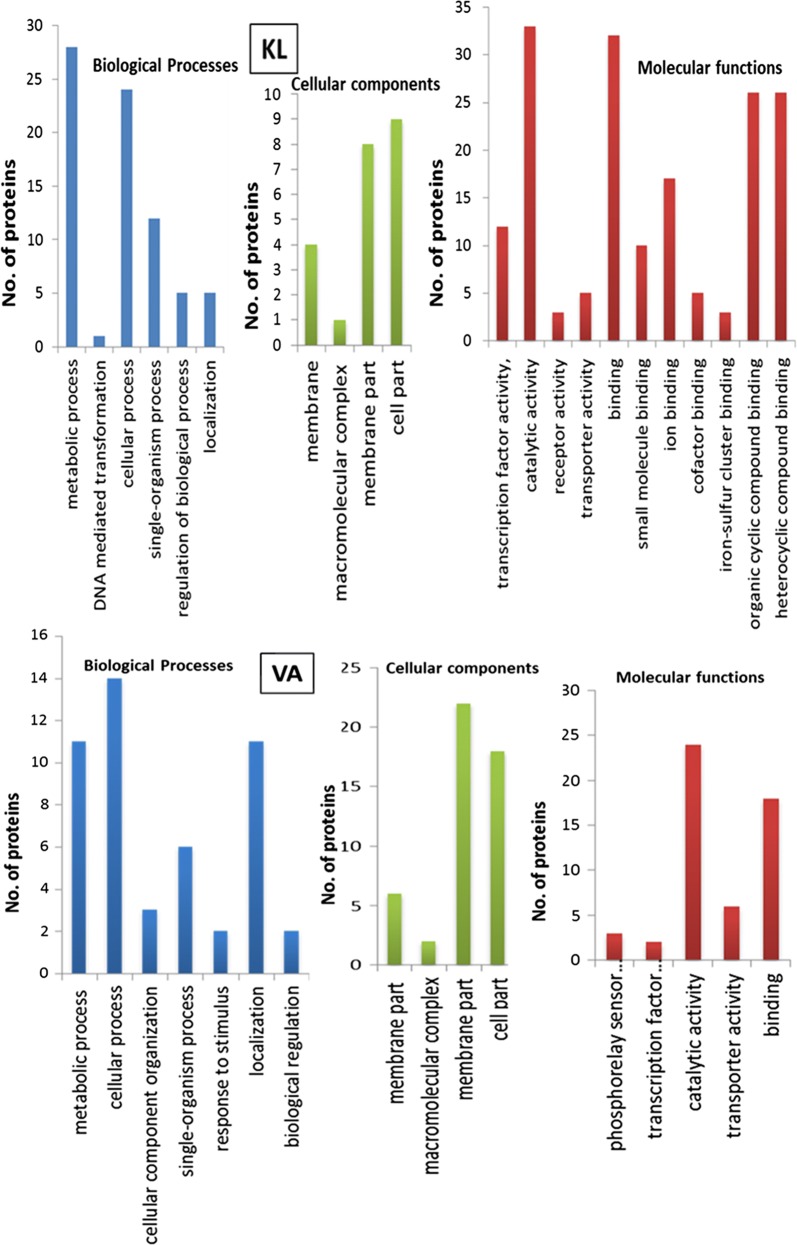
GO analysis of protein expressed by *Pandoraea* sp. ISTKB while growing on KL and VA. The expressed proteins were classified into biological processes, cellular components, and molecular functions

The GO analysis of genomics was supported by proteomics (especially biological processes and molecular functions) on KL and VA (Fig. 8). The molecular functions category indicates an abundance of protein in catalytic activity, heterocyclic compound binding, organic compound binding, and transcription factor activity on KL and absent on VA. Single organism process was found to be dominant in KL and VA (after normal cellular and metabolic processes) indicates this strain specific process. The proteins involved in localization process on VA were almost double compared to KL. The membrane protein was present in KL and VA, but their representation on VA was found to be more than double as compared to KL and the transporters were also expressed more in VA.

### Expressed proteins involved in lignin or aromatic compound degradation

Proteomic profile of *Pandoraea* sp. ISTKB revealed the presence of relevant proteins expressed only on KL or VA (Table 1) and KL–VA, as represented in Tables 2, 3, and 4. There are 17, 29, and 394 uncharacterized proteins observed in the KL, VA, and KL–VA, respectively. The various functionally active oxidoreductases, methyltransferases, hydrolases, isomerases, dehydrogenases, reductases, transferases, esterases, transporters, transcriptional factors, stress response, and detoxification-related proteins were observed that could play important role in degradation of lignin or aromatic compounds.

**Table 1:** Identification of relevant proteins expressed only on kraft lignin (KL) or vanillic acid (VL) that can assist in lignin degradation

Uniprot entry	Gene locus tag	Protein names	LFQ intensity KL	Razor + unique peptides KL	Sequence coverage (%)	Mol. weight (kDa)	Intensity
Relevant protein expressed only on kraft lignin (KL)							
A0A1E3LHD6	A9762_20370	Tryptophan 2,3-dioxygenase	22.9781	2	12.3	36.655	0.00043
A0A1E3LB56	A9762_07750	Benzoyl-CoA oxygenase subunit B	28.4307	15	41.7	54.265	2.06E−87
A0A1E3LET3	A9762_23815	Acriflavine resistance protein	22.5124	1	1.2	112.36	0.00162
A0A1E3LL12	A9762_14780	Glycine betaine ABC transporter substrate-binding protein	23.8554	2	6.2	36.204	0.00036
A0A1E3LI04	A9762_22630	Enoyl-CoA hydratase	23.5827	2	13.3	28.399	1.38E−14
A0A1E3LPU3	A9762_13345	Pyruvate ferredoxin oxidoreductase	17.3225	3	4	129.05	0.00016
A0A1E3LLU4	A9762_13050	Carboxyvinyl-carboxyphosphonate phosphorylmutase	24.4057	2	14.3	31.557	0.00039
A0A1E3LBB9	A9762_07755	Benzoyl-CoA oxygenase/reductase, BoxA protein	23.9233	2	5	45.826	5.25E−07
A0A1E3LGI4	A9762_03990	SAM-dependent methyltransferase	24.6086	2	9.6	31.525	1.73E−06
A0A1E3LDW7	A9762_25245	(2Fe–2S)-binding protein	24.4339	2	24.5	20.242	2.06E−06
A0A1E3LNU5	A9762_10215	LysR family transcriptional regulator	23.5144	2	8.4	33.65	4.15E−07
A0A1E3LEP7	A9762_23880	Phenylacetic acid degradation protein	24.5346	3	12.4	39.494	4.34E−14
A0A1E3LJ38	A9762_17050	ABC transporter	26.2152	5	18.3	32.972	3.46E−34
A0A1E3LB77	A9762_07880	ABC transporter ATP-binding protein	24.5787	2	9.7	25.723	2.69E−12
A0A1E3LF42	A9762_23860	1,2-Phenylacetyl-CoA epoxidase subunit A (monooxygenase)	25.5084	4	14	37.739	4.64E−20
A0A1E3LGK2	A9762_23865	1,2-Phenylacetyl-CoA epoxidase subunit B (monooxygenase)	24.5067	2	20.4	11.224	6.77E−06
A0A1E3LHG4	A9762_23220	Formyl-CoA:oxalate CoA transferase	26.7341	5	17.8	45.737	4.76E−34
A0A1E3LNE1	A9762_10935	Salicylate hydroxylase	22.5412	1	3.1	41.287	0.00183
A0A1E3LF93	A9762_23590	Ligand-gated channel protein	24.4315	2	3.4	81.344	8.95E−05
A0A1E3LHJ6	A9762_19845	NADPH:quinone reductase	23.3346	1	3.1	31.317	0.00029
A0A1E3LIQ8	A9762_17970	Glycolate oxidase subunit GlcE	24.8966	2	7.3	40.542	3.71E−10
A0A1E3LEZ8	A9762_23215	2-Hydroxyhepta-2,4-diene-1,7-dioate isomerase	24.1273	2	14.8	27.8	1.39E−06
Relevant protein expressed only on vanillic acid (VA)							
A0A1E3LRS2	A9762_00545	Alkene reductase	26.4047	7	29.1	39.612	3.91E−37
A0A1E3LLI9	A9762_02605	Alpha/beta hydrolase	25.8742	4	23.2	30.983	3.92E−11
A0A1E3LLX9	A9762_03340	Tol-pal system-associated acyl-CoA thioesterase	24.2249	2	14.4	17.548	2.38E−05
A0A1E3LDT8	A9762_25265	Acetyltransferase	24.2249	2	14.4	17.548	2.38E−05
A0A1E3LPL0	A9762_01420	Glutathione *S*-transferase	24.4839	2	15.3	24.629	2.35E−07
A0A1E3LCR5	A9762_26030	Aminomethyltransferase	25.197	2	11	34.276	8.63E−05
A0A1E3LPI9	A9762_13065	Methyltransferase	22.991	2	7.7	29.85	1.53E−06
A0A1E3LEN5	A9762_06460	Rieske (2Fe–2S) protein	25.0064	1	6.5	43.065	1.25E−08
A0A1E3LHR2	A9762_19260	Glycine/betaine ABC transporter permease	24.0824	1	4.9	25.659	0.0001153

**Table 2:** Differentially expressed proteins for phenylacetic acid, benzene degradation, and various oxidoreductases on kraft lignin

Uniprot IDs	Locus tag	Name	Log2 fold change	Unique peptides	Sequence coverage (%)	Mol. weight (kDa)	Intensity
		*Phenylacetic acid degradation protein*					
A0A1E3LF26	A9762_23720	Phenylacetic acid degradation protein PaaD	0.529261	3	27.3	15.022	8.65E−26
A0A1E3LF48	A9762_23735	Phenylacetic acid degradation protein PaaN	0.244067	18	58.3	60.008	1.24E−140
A0A1E3LFB3	A9762_23725	2-(1,2-Epoxy-1,2-dihydrophenyl)acetyl-CoA isomerase	0.724358	9	56.1	27.854	1.32E−76
A0A1E3LFJ2	A9762_22495	Phenylacetic acid degradation protein	0.790541	4	36.3	15.888	3.07E−15
A0A1E3LHE3	A9762_23715	Phenylacetate–coenzyme A	0.514503	14	57.5	47.386	1.43E−109
A0A1E3LQE8	A9762_10500	Phenylacetic acid degradation protein	1.426558	5	44.4	14.521	5.16E−22
		*Peroxidases*					
A0A1E3LDX8	A9762_24250	Dyp-type peroxidase	1.43239	15	72	40.756	1.29E−104
A0A1E3LHN8	A9762_20355	Peroxidase	− 1.69967	18	90.6	23.753	0
A0A1E3LPA6	A9762_00985	Chloroperoxidase	− 1.927944	16	84.7	30.075	8.73E−175
A0A1E3LF97	A9762_25345	Peroxidase-like protein	1.353771	7	59	18.981	4.85E−31
A0A1E3LNE3	A9762_13620	Laccase	− 0.13346	10	57.5	28.644	5.45E−43
		*Oxidases*					
A0A1E3LC86	A9762_26490	Glycolate oxidase subunit GlcE	1.96696	9	38.1	38.774	4.18E−40
A0A1E3LDU3	A9762_25250	Aldehyde oxidase	1.94888	25	43.2	106.43	3.40E−153
A0A1E3LG04	A9762_25240	Cytochrome C oxidase Cbb3	1.88749	12	44.8	44.919	5.64E−68
A0A1E3LL61	A9762_17965	Glycolate oxidase iron–sulfur subunit	1.88269	5	14.3	46.4	1.56E−20
A0A1E3LCC6	A9762_07195	Oxidase	1.12594	23	67.7	43.498	4.04E−279
A0A1E3LQ45	A9762_00290	FAD-linked oxidase	0.7576	46	47.6	148.68	8.22E−260
A0A1E3LDS7	A9762_25555	Ubiquinol oxidase subunit 2	− 0.72481	3	21.5	35.767	2.50E−21
A0A1E3LLY2	A9762_16250	l-Aspartate oxidase	− 1.51593	5	14.2	58.534	1.25E−24
A0A1E3LRG9	A9762_02095	Cytochrome c oxidase assembly protein	− 1.76256	6	49.8	22.192	2.52E−27
A0A1E3LCR8	A9762_26505	2-Hydroxy-acid oxidase	− 1.92172	13	43.3	51.242	1.38E−99
		*Oxidoreductases*					
A0A1E3L9T7	A9762_09290	NADH–quinone oxidoreductase subunit I	1.983	13	54.6	18.63	1.26E−66
A0A1E3LC99	A9762_26255	Oxidoreductase	1.46342	9	62.5	26.21	1.99E−100
A0A1E3LAC9	A9762_09275	NADH oxidoreductase (quinone) subunit F	0.86275	19	70.7	47.093	5.72E−135
A0A1E3LMK8	A9762_03120	NADP oxidoreductase	0.82498	10	55.5	32.458	3.02E−38
A0A1E3LHK5	A9762_23470	FAD-dependent oxidoreductase	0.33833	3	14.7	38.458	4.28E−15
A0A1E3LLW4	A9762_03220	GMC family oxidoreductase	0.24372	23	54.8	64.908	3.74E−163
A0A1E3LFG3	A9762_22500	NADP-dependent oxidoreductase	0.05857	13	63.4	35.64	5.98E−106
A0A1E3LKB2	A9762_03335	Oxidoreductase	− 0.24038	18	82.3	31.785	3.07E−157
A0A1E3LGB9	A9762_21450	YggW family oxidoreductase	− 0.68269	3	10.3	45.723	1.71E−14
A0A1E3LC87	A9762_26930	Fe–S oxidoreductase	− 1.47441	6	49.8	26.119	2.01E−34
A0A1E3LG91	A9762_21265	Vanillate *O*-demethylase ferredoxin subunit	− 1.5017	15	57.9	33.723	1.13E−114
A0A1E3LG83	A9762_21255	Vanillate *O*-demethylase oxidoreductase	0.298001	15	48.1	50.745	1.80E−76
A0A1E3LFZ4	A9762_25310	FAD-dependent oxidoreductase	− 1.67016	5	17.9	54.42	2.53E−15
A0A1E3LA58	A9762_09270	NADH-quinone oxidoreductase subunit E	− 1.69506	9	80.1	18.129	9.41E−90
A0A1E3LFR2	A9762_22175	Oxidoreductase	− 1.8627	9	61.8	30.679	4.89E−178
A0A1E3LCB2	A9762_09255	NADH-quinone oxidoreductase subunit B	− 1.96385	10	72.3	17.519	1.56E−64
A0A1E3LEJ8	A9762_05735	Oxidoreductase	− 1.97509	18	85.7	26.372	6.17E−220
A0A1E3LKX1	A9762_15740	Quinone oxidoreductase	− 1.99406	18	90.7	34.556	1.06E−219
		*Oxygenases*					
A0A1E3LHN5	A9762_23090	2-Nitropropane dioxygenase	1.84558	3	15.1	38.789	7.50E−15
A0A1E3LFU3	A9762_22350	Quercetin 2,3-dioxygenase	1.82796	7	51.5	26.31	4.21E−24
A0A1E3LFX1	A9762_21815	Homogentisate 1,2-dioxygenase	1.51818	13	56.9	48.611	5.10E−81
A0A1E3LPA9	A9762_00970	4-Hydroxyphenylpyruvate dioxygenase	1.43964	2	5.9	40.191	3.14E−09
A0A1E3LFB4	A9762_23095	2-Nitropropane dioxygenase	1.31977	9	45.1	39.332	6.27E−61
A0A1E3LDU6	A9762_24595	Phytanoyl-CoA dioxygenase	− 0.60384	6	38.3	27.846	2.72E−52
A0A1E3LF51	A9762_25450	Putative dioxygenase	− 0.666263	9	88.7	15.676	1.86E−122
A0A1E3LIM3	A9762_17955	Dioxygenase	− 0.91661	8	52	29.958	6.54E−31
A0A1E3LI70	A9762_22255	2-Nitropropane dioxygenase	− 1.45193	12	64.4	33.509	1.71E−146
A0A1E3LIR0	A9762_17450	Protocatechuate 3,4-dioxygenase subunit alpha	− 1.63442	13	83.4	21.895	2.50E−246
A0A1E3LJ07	A9762_17445	Protocatechuate 3,4-dioxygenase subunit beta	− 1.71052	16	78.4	26.513	2.43E−152
A0A1E3LG93	A9762_21285	Protocatechuate 4,5-dioxygenase subunit alpha	− 1.83931	6	80.7	13.769	6.11E−47
A0A1E3LG93	A9762_21285	Protocatechuate 4,5-dioxygenase subunit beta	− 1.83931	6	80.7	13.769	6.11E−47
A0A1E3LEL8	A9762_04935	Antibiotic biosynthesis monooxygenase	− 1.86712	6	81.8	11.026	1.02E−172
A0A1E3LBV9	A9762_07570	2OG-Fe(II) oxygenase	− 1.682965	7	50.7	30.695	9.21E−31
A0A1E3LL67	A9762_14910	2OG-Fe(II) oxygenase	− 0.037474	8	43	37.152	3.47E−45
		*Benzoate degradation*					
A0A1E3LBS0	A9762_08405	2-Aminobenzoate–CoA ligase	1.569646	5	15.2	59.58	4.06E−18
A0A1E3LF67	A9762_23480	3-Octaprenyl-4-hydroxybenzoate carboxy-lyase (Fragment)	− 1.471879	4	32.4	15.528	1.01E−27
A0A1E3LLJ4	A9762_17745	3-Octaprenyl-4-hydroxybenzoate carboxy-lyase	1.567849	11	32.8	57.345	1.54E−68
A0A1E3LM76	A9762_12465	2-Nonaprenyl-3-methyl-6-methoxy-1,4-benzoquinol hydroxylase	− 1.895105	5	47.4	23.511	1.50E−34
A0A1E3LDW9	A9762_24905	Carboxymethylenebutenolidase	− 1.707803	8	49	27.008	1.57E−119
A0A1E3LDA6	A9762_06440	Carboxymethylenebutenolidase	− 1.951469	18	77	31.135	1.41E−118

**Table 3:** Differentially expressed antioxidant and stress response proteins on kraft lignin

Uniprot IDs	Locus tag	Name	Log2 fold change	Unique peptides	Sequence coverage (%)	Mol. weight (kDa)	Intensity
		*Glutathione enzymes*					
A0A1E3LF33	A9762_23395	Glutathione ABC transporter substrate-binding protein	1.80871	29	77.1	57.261	0
A0A1E3LKY8	A9762_17040	Glutathione *S*-transferase	1.18746	8	41.1	24.027	3.57E−36
A0A1E3LF68	A9762_25435	Glutathione *S*-transferase	0.61286	8	65.2	22.821	1.27E−72
A0A1E3LBR6	A9762_09395	Glutathione *S*-transferase	0.55477	4	25.7	24.666	5.23E−14
A0A1E3LQD9	A9762_10375	Glutathione *S*-transferase	0.51263	5	37.8	27.794	1.84E−17
A0A1E3LFJ0	A9762_24585	Glutathione-disulfide reductase	0.35879	22	63.6	48.837	7.37E−222
A0A1E3LI91	A9762_18915	Glutathione *S*-transferase	0.05437	5	39	25.252	2.31E − 26
A0A1E3LP56	A9762_00695	Glutathione *S*-transferase	− 0.96784	8	53.6	23.768	4.31E−48
A0A1E3LI39	A9762_18265	Glutathione *S*-transferase	− 1.44982	18	82.3	23.728	3.01E−285
A0A1E3LAC5	A9762_09365	Glutathione *S*-transferase	− 1.70035	15	72.6	26.078	1.22E−149
A0A1E3LPY3	A9762_02245	Glutathione synthetase	− 1.79520	21	82.4	34.566	2.19E−195
A0A1E3LL68	A9762_15125	Glutathione *S*-transferase	− 1.85572	4	21	23.996	5.81E−18
A0A1E3LI75	A9762_04000	Lactoylglutathione lyase	− 1.97244	4	64.5	14.032	1.22E−30
A0A1E3LBC2	A9762_08325	Hydroxyacylglutathione hydrolase	− 1.98082	10	57.5	29.087	5.85E−75
A0A1E3LC63	A9762_26475	Glutathione peroxidase	1.59171	9	82.6	18.506	1.18E−57
A0A1E3LN82	A9762_11030	Glutathione peroxidase	− 1.47148	10	55.2	19.852	3.04E−128
A0A1E3LL32	A9762_17770	*S*-(Hydroxymethyl)glutathione dehydrogenase	− 1.97881	22	83.7	39.609	5.20E−235
A0A1E3LCG8	A9762_26430	*S*-Formylglutathione hydrolase	− 1.5565	9	49.6	31.49	2.83E−45
		*Catalases*					
A0A1E3LJG2	A9762_17205	Catalase	− 1.90503	31	69.7	55.065	0
A0A1E3LHV5	A9762_19890	Catalase	1.96803	20	58.3	54.314	2.70E−159
A0A1E3LL41	A9762_15065	Catalase	1.96803	20	58.3	54.314	2.70E−159
		*Superoxide dismutase*					
A0A1E3LHJ2	A9762_20590	Superoxide dismutase	− 1.97424	2	12.2	22.201	8.04E−07
A0A1E3LJK7	A9762_16420	Superoxide dismutase	− 1.6319	16	93.2	21.3	0
		*Thioredoxin*					
A0A1E3LA95	A9762_09775	Thioredoxin	1.50102	6	68.5	11.693	1.86E−50
A0A1E3LIM9	A9762_21500	Thioredoxin	0.26594	13	64	30.297	2.12E−120
A0A1E3LMC9	A9762_12720	Probable thiol peroxidase	− 1.29118	15	95.8	17.552	1.19E−229
A0A1E3LK52	A9762_19935	Thioredoxin reductase	− 0.42918	12	71.1	33.796	6.61E−169
		*Peroxiredoxin*					
A0A1E3LFM2	A9762_22670	Peroxiredoxin	0.06241	4	27.7	19.976	2.59E−09
A0A1E3LG33	A9762_25080	Peroxiredoxin	− 0.7565	5	51.9	17.411	3.34E−34
A0A1E3LIX5	A9762_17525	Peroxiredoxin	1.63617	11	84.5	20.829	4.52E−87
A0A1E3LNW8	A9762_00130	Peroxiredoxin	− 1.41372	5	35	14.926	1.51E−22
		*Glyoxylase*					
A0A1E3LJS5	A9762_02855	Glyoxalase	1.90407	4	26.4	24.933	1.26E−10
A0A1E3LML8	A9762_12845	Glyoxalase	− 1.55877	4	40.7	15.609	4.05E−12
		*Glutaredoxin*					
A0A1E3LF15	A9762_23485	Glutaredoxin	− 0.65795	4	51.9	11.612	1.33E−69
A0A1E3LQ32	A9762_02205	Glutaredoxin 3	1.02376	8	79.1	9.8904	4.95E−60
		*Alkylperoxide reductase*					
A0A1E3LAL5	A9762_08705	Alkyl hydroperoxide reductase	− 1.92917	10	79.1	16.924	2.59E−121
A0A1E3LDK0	A9762_25350	Alkyl hydroperoxide reductase AhpD	1.76395	5	40.7	22.242	5.04E−30
A0A1E3LCA5	A9762_26215	Alkyl hydroperoxide reductase AhpD	− 1.58398	5	49.5	19.7	1.06E−56
A0A1E3LGT9	A9762_04630	Alkyl hydroperoxide reductase AhpD	− 0.87073	6	67.7	14.207	2.45E−17
A0A1E3LKG5	A9762_16100	Alkyl hydroperoxide reductase AhpD	− 1.52586	9	64.9	21.888	2.68E−61
A0A1E3LLQ8	A9762_13730	Alkyl hydroperoxide reductase AhpD	− 0.29909	10	77.7	18.588	4.23E−101
A0A1E3LLX0	A9762_13735	Alkyl hydroperoxide reductase	− 0.30453	16	85.7	20.001	0

**Table 4:** Differentially expressed reductase, dehydrogenase, transferase, and hydratase proteins on kraft lignin

Uniprot IDs	Locus tag	Name	Log2 fold change	Unique peptides	Sequence coverage (%)	Mol. weight (kDa)	Intensity
		*Reductases*					
A0A1E3LM36	A9762_03695	Aldo/keto reductase	− 1.446536	54	95.1	38.448	0
A0A1E3LPJ8	A9762_01385	Aldo/keto reductase	− 1.811371	8	43.3	37.797	1.87E−50
A0A1E3LS07	A9762_01040	Aldo/keto reductase	0.040585	20	73.6	30.685	1.63E−183
A0A1E3LG16	A9762_22220	Glyoxylate/hydroxypyruvate reductase A	1.99991	14	69.4	33.941	4.47E−124
A0A1E3LQJ8	A9762_00050	Bifunctional glyoxylate/hydroxypyruvate reductase B	− 0.601011	20	82.2	34.525	1.34E−204
A0A1E3LI17	A9762_19295	Alkene reductase	− 0.075801	28	92	40.61	0
A0A1E3LIA9	A9762_18295	2-Alkenal reductase	0.117504	12	46.4	42.805	2.10E−74
A0A1E3LDX7	A9762_24675	Ferredoxin–NADP(+) reductase	1.250238	18	77	29.272	3.54E−106
A0A1E3LFT3	A9762_21855	NADPH-dependent FMN reductase	− 0.973988	9	69.1	19.818	1.98E−77
A0A1E3LAB2	A9762_08440	NADPH:quinone reductase	− 1.850827	14	71.9	36.075	3.86E−109
A0A1E3LQJ8	A9762_00050	Hydroxypyruvate reductase B	− 0.601011	20	82.2	34.525	1.34E−204
A0A1E3LLZ7	A9762_13130	Fumarate reductase	1.805886	2	7.4	52.225	7.89E−05
		*Dehydrogenases*					
A0A1E3LDJ7	A9762_25850	Formate dehydrogenase subunit beta	1.664541	6	23.2	34.213	1.25E−22
A0A1E3LDC4	A9762_25845	Formate dehydrogenase-*N* subunit alpha	1.414862	16	34.2	90.668	3.91E−102
A0A1E3LDS6	A9762_24610	Formate dehydrogenase	0.761355	2	57.1	8.7799	3.84E−22
A0A1E3LE53	A9762_24625	Formate dehydrogenase	1.257519	9	30	56.957	3.45E−62
A0A1E3LG97; A0A1E3LF08	A9762_24620	Formate dehydrogenase subunit alpha	1.020855	21	34	104.98	1.29E−121
A0A1E3LL63	A9762_15395	NADH dehydrogenase	0.064345	9	60.8	21.897	5.70E−49
A0A1E3LPJ3	A9762_01450	Aldehyde dehydrogenase	1.745055	17	54.5	52.735	1.72E−105
A0A1E3LJ61	A9762_20360	Acyl-CoA dehydrogenase	0.210716	11	46.1	43.33	1.22E−90
A0A1E3LH50	A9762_22885	Alcohol dehydrogenase	0.448181	14	55.4	40.556	1.68E−67
A0A1E3LGE7	A9762_21150	Aldehyde dehydrogenase	0.513856	4	14.4	51.073	1.64E−12
A0A1E3LFY2	A9762_21880	NAD(FAD)-utilizing dehydrogenase	1.806462	3	12.9	42.366	7.90E−09
A0A1E3LF76	A9762_23430	Acyl-CoA dehydrogenase	0.067218	9	31.6	40.901	1.33E−56
A0A1E3LKN0	A9762_15340	Acyl-CoA dehydrogenase	− 1.0527852	7	24.4	41.792	1.04E−41
A0A1E3LP12	A9762_00210	Acyl-CoA dehydrogenase	− 0.467758	37	72.4	65.029	0
A0A1E3LKM0	A9762_16035	NAD(P)H dehydrogenase (quinone)	− 1.786348	11	55.7	22.107	2.74E−162
A0A1E3LKB6	A9762_03215	Alcohol dehydrogenase	− 1.58185	16	63.3	44.426	1.36E−99
A0A1E3LJV2	A9762_02775	Putative NADH dehydrogenase	− 0.256436	15	86.3	21.594	1.71E−184
A0A1E3LIB8	A9762_18460	Short-chain dehydrogenase	− 1.862879	2	13.3	26.395	8.23E−08
A0A1E3LI49	A9762_22120	Short-chain dehydrogenase	− 1.335172	6	29.3	24.127	2.78E−23
A0A1E3LI38	A9762_22070	Short-chain dehydrogenase	− 1.826239	3	15.2	29.599	1.04E−17
A0A1E3LHQ0	A9762_20005	Dehydrogenase	− 0.022626	13	53.6	38.384	2.57E−91
A0A1E3LHP2	A9762_20015	Aldehyde dehydrogenase	− 0.39059	15	63.3	51.925	2.00E−131
A0A1E3LHJ3	A9762_19710	Short-chain dehydrogenase	− 0.299807	5	32.7	26.91	1.68E−19
A0A1E3LLS1	A9762_17065	Short-chain dehydrogenase	− 1.1228889	10	50.4	29.165	2.51E−85
A0A1E3LFZ7	A9762_24160	Acyl-CoA dehydrogenase	− 1.977129	8	13.2	90.723	3.27E−24
A0A1E3LFV1	A9762_21935	Alcohol dehydrogenase	− 0.223075	15	71.1	36.536	2.10E−159
A0A1E3LF72	A9762_23085	NADPH:quinone dehydrogenase	− 1.970038	12	66.5	34.703	2.88E−132
A0A1E3LE86	A9762_05015	Short-chain dehydrogenase	− 0.122157	19	90.8	26.28	1.25E−173
A0A1E3LE78	A9762_24110	Short-chain dehydrogenase	− 1.0213605	8	59.1	24.582	6.63E−39
A0A1E3LE10	A9762_24765	Aldehyde dehydrogenase	− 0.713344	26	69.6	50.552	2.84E−236
A0A1E3LDF9	A9762_25465	Short-chain dehydrogenase	− 1.0369092	18	82.1	26.766	0
A0A1E3LCV6	A9762_26070	Acyl-CoA dehydrogenase	− 0.559374	33	78.2	63.6	0
A0A1E3LCK3	A9762_26745	Aldehyde dehydrogenase	− 1.998458	41	82.4	55.098	0
A0A1E3LCD2	A9762_09370	Acyl-CoA dehydrogenase	− 0.349182	10	31.9	45.179	6.63E−43
		*Transferase and hydratase*					
A0A1E3LDE2	A9762_25255	Acetyltransferase	1.1424203	5	42.2	19.294	9.45E−19
A0A1E3LEG1	A9762_24350	Acyl-CoA transferase	− 1.894681	5	23.8	49.528	1.44E−09
A0A1E3LE25	A9762_26525	Acyltransferase	− 0.658173	11	53.7	31.029	2.25E−127
A0A1E3LCY1	A9762_06345	CoA transferase	− 1.644591	5	19.7	43.746	5.43E−17
A0A1E3LAQ8	A9762_09850	Formyl-CoA transferase	1.63564	16	57	43.01	7.70E−140
A0A1E3LEY2	A9762_23230	Formyl-CoA:oxalate CoA transferase	1.480644	23	65.9	45.431	4.87E−142
A0A1E3LF20	A9762_25975	*N*-Hydroxyarylamine *O*-acetyltransferase	− 0.812216	13	68.8	31.71	1.16E−71
A0A1E3LB74	A9762_08395	Enoyl-CoA hydratase	0.844962	4	17.4	30.92	1.3832E−11
A0A1E3LG06	A9762_22140	Enoyl-CoA hydratase	− 0.845624	7	39.9	28.125	5.7526E−122
A0A1E3LD66	A9762_27340	Enoyl-CoA hydratase	1.887871	9	53.1	29.49	1.5759E−26
A0A1E3LNX6	A9762_00200	Acetyl-CoA acetyltransferase	− 0.329002	26	92.5	41.664	0
A0A1E3LED2	A9762_24145	Acetyl-CoA acetyltransferase	− 0.647329	11	34.2	46.667	5.8563E−44
A0A1E3LFL9	A9762_22115	Acetyl-CoA acetyltransferase	− 1.992637	21	75.9	40.895	0
A0A1E3LLK2	A9762_13635	Acetyl-CoA acetyltransferase	− 1.672549	24	85	40.687	0
A0A1E3LLN7	A9762_13660	Acetyl-CoA acetyltransferase	− 1.969308	20	70.5	41.273	0
A0A1E3LFA3	A9762_23740	Enoyl-CoA hydratase	− 1.935441	19	86.8	28.063	8.927E−161
A0A1E3LNG2	A9762_11320	Enoyl-CoA hydratase	0.497127	6	36.4	27.805	7.4808E−33
A0A1E3LNH0	A9762_11445	Enoyl-CoA hydratase	− 1.83413	8	45.4	29.067	2.3968E−55
A0A1E3LNY9	A9762_00195	Enoyl-CoA hydratase	− 0.4152	17	84.7	27.622	1.6359E−146

### Important proteins expressed either on kraft lignin or on vanillic acid

The analysis of expression profile on KL revealed the presence of 1,2-phenylacetyl-CoA epoxidase (monooxygenase), phenylacetic acid degradation protein, and 2-hydroxyhepta-2,4-diene-1,7-dioate isomerase enzymes for the degradation of phenylacetate. Proteins such as benzoyl-CoA oxygenase, enoyl-CoA hydratase, tryptophan 2,3-dioxygenase, and salicylate hydroxylase were also active on KL. Proteins for methyl group transfer and decarboxylation such as SAM-dependent methyltransferase, pyruvate ferredoxin oxidoreductase, and (2Fe–2S)-binding protein were also observed. Generation of reactive intermediates and their detoxification by oxidative stress-resistance protein glycolate oxidase and NADPH:quinone reductase was present. Glycine betaine ABC transporter substrate-binding protein and formyl-CoA:oxalate CoA-transferase (FCOCT) proteins for osmoprotection and acid response regulator were present to maintain the smooth functioning of intracellular environment. There were six LysR family, two unclassified and one each of GntR family, AsnC family, Cd(II)/Pb(II)-responsive, Crp/Fnr family, MarR, and MerR transcriptional regulator found on KL. The VA was mainly dominated by transporters and stress response proteins [glutathione *S*-transferase, Rieske (2Fe–2S) protein, thioesterase, glycine betaine permease, and alkene reductase]. One methyltransferases, aminomethyltransferase, and LysR family transcriptional regulator were also observed.

### Proteins differentially expressed on kraft lignin and vanillic acid

There were 1979 proteins obtained on KL–VA after normalization, and among these, 1110 proteins upregulated and 869 downregulated on kraft lignin. There are 164 transporters detected out of which 127 are ABC, 5 RND, and 4 MFS. There are 163 transcription factors identified comparising 34 LysR family, 21 GntR family, 17 tetR family, 12 each MarR, and IcIR family. We are discussing here important proteins that can perform lignin degradation and transformation. Some of the differentially expressed proteins that may involve in prospective lignin degradation are shown in Fig. 7b. The presence of various oxidoreductases, dehydrogenase, reductases, transferases, PHA biosynthetic proteins, and several stress response and detoxification proteins was detected in the expression profile. The phenylacetic acid degradation protein and ‘CoA’-mediated degradation of phenylacetate, phenylpropionate, and benzoate proteins were found to be upregulated on kraft lignin. The DyP-type peroxidase, peroxidase-like proteins, and various accessory enzymes such as aldehyde oxidase, glycolate oxidase, cytochrome C oxidase, oxidase, NADH:quinone oxidoreductase, FAD-linked oxidase, and GMC family oxidoreductase were found to be upregulated on KL. GMC family oxidoreductase or aryl alcohol oxidase is also known as auxiliary enzymes in case of fungi and their role is established in lignin degradation [27]. The homogentisate 1,2-dioxygenase, quercetin 2,3-dioxygenase, 4-hydroxyphenylpyruvate dioxygenase, dioxygenase, and nitropropane dioxygenase were found to be upregulated on KL. There were six SAM-dependent methyltransferase and one methyltransferase identified on KL–VA. Four SAM-dependent methyl transferase and methyltransferase was upregulated on KL and two SAM-dependent methyltransferase was upregulated on VA.

The expression of antioxidant and stress response proteins glutathione peroxidase, glutathione-disulfide reductase, catalase, glyoxylase, thioredoxin, peroxiredoxin, alkyl hydroperoxide reductase, aldo/keto reductase, and glutathione *S*-transferases was upregulated in case of KL. Superoxide dismutase was downregulated in case of KL and catalases were downregulated on VA. The proteins formyl-coA transferase, formate dehydrogenase for oxalate, and formate metabolism were also found to be upregulated on KL. Various other dehydrogenases, reductases, and transferases such as hydroxypyruvate reductase, NAD dehydrogenase, alcohol dehydrogenase, aldehyde dehydrogenase, ferredoxin reductase, ferredoxin, acyl-CoA dehydrogenase, acetyltransferases, and enoyl-CoA hydratase, were upregulated on KL.

The expression of vanillate O-demethylase oxidoreductase, chloroperoxidase, hydroglutathione hydrolase, protocatechuate 3,4-dioxygenase, protocatechuate 4,5-dioxygenase, 2OG-Fe(II) oxygenase, antibiotic synthesis monooxygenase, 2-hydroxyl acid oxidase, cytochrome c oxidase, NADH quinone oxidoreductase, glutathione peroxidase, and other oxidoreductases was upregulated in case of VA. The expression of protocatechuate 4,5-dioxygenase was more than double compared to protocatechuate 3,4-dioxygenase on VA. Compared to KL, the expression of oxidases enzymes was very less on VA. The expression of laccase, FAD-dependent oxidoreductase, phytanoyl-CoA dioxygenase, YggW family oxidoreductase, ubiquinol oxidase, one glutathione *S*-transferase, and NADH quinone oxidoreductase, was almost same in both KL and VA. There were several NADH:quinone oxidoreductases observed in KL–VA and some are upregulated in KL other in VA. Short-chain dehydrogenase, acyl-CoA dehydrogenase, alcohol dehydrogenase, acyltransferase, alkene reductase, FMN reductase, NADH:quinone reductase, and acetyl-CoA acetyl transferase was found to be upregulated on VA.

The clusters predicted for lignin degradation and PHA production were found to functionally active and the genes for degradation of lignin derivatives as well as all the three PHA polymerase were present in the expression profile (Additional file 3: Table S10, also contains other dehydrogenase, reductases, transferases, esterases, thioesterases, hydrolases not discussed here but expressed on KL–VA). The PHA production was induced on both the substrate, i.e., kraft lignin and vanillic acid. The activation of PHA biosynthetic genes on lignin was also recently reported [17].

## Discussion

The detail of genomic and proteomic studies of lignin degrading bacterium is limited, so we tried to provide the comprehensive genomic and proteomic analysis of lignin degrading bacterium *Pandoraea* sp. ISTKB. The genome size of this genus available in NCBI varies between 4.4 and 6.5 Mb and this strain’s genome is one of the largest genome sequences available until date from *Pandoraea* genus. The degradation of aromatic compounds by bacteria is mostly aerobic and is tightly regulated process. Their degradation by oxidoreductases generates reactive intermediates, so a robust stress response and detoxification mechanism is required for survival of microbes. The dominance of these subsystem features such as respiration, aromatic metabolism, and stress response (after normal cellular processes) and their complementation highlights the ability of *Pandoraea* sp. ISTKB to survive and metabolize lignin or aromatic compound.

The GO analysis especially biological process and molecular functions indeed supported this strain’s robust genomic machinery for the utilization of organic substance, organic cyclic compounds, heterocyclic compound binding, solute binding, ion binding, and oxidoreductase activity. The abundance of localization process proteins, membrane proteins, and transporters in VA as compared to KL can be explained that these proteins might be localized near the membrane and actively involved in transportation and metabolism of VA into the cell. The absence of proteins in VA for organic cyclic compound binding, heterocyclic compound binding, iron–sulfur cluster binding, receptor activity, ion binding, cofactor binding, small molecule binding, and their presence in KL suggests that these are the important molecular functions’ category proteins that would have facilitated the depolymerization and utilization of polymer KL by this strain.

The analysis of expression profile on KL indicates the presence of metacleavage and unusual pathways, i.e., ‘-CoA’-mediated degradation of lignin derivatives in aerobic microorganisms. The presence of 2-hydroxyhepta-2,4-diene-1,7-dioate isomerase in the expression profile of KL possibly indicated 4-hydroxyphenylacetate degradation through meta cleavage pathways [28]. Benzoyl-CoA oxygenase-mediated degradation of aromatic compound is completely different mechanisms and observed in 4–5% of sequenced bacterial genomes. This mechanism helps to overcome the high resonance stabilization of aromatic ring by forming epoxide. Benzoyl-CoA oxygenase leads to formation of 2,3-epoxide followed by enoyl-CoA hydratase (also expressed on KL) and NADP^+^-dependent aldehyde dehydrogenase (upregulated on KL)-mediated degradation resulting into formic acid, acetyl-CoA, and succinyl-CoA formation [29]. 1,2-phenylacetyl-CoA epoxidase-mediated degradation of phenylacetic acid occurs via 1,2-epoxide intermediate and this pathway is found functional in only 16% of all bacteria genome reported also observed in *Escherichia coli* and *Pseudomonas putida* [30]. The upregulation of *Salicylate* hydroxylase on lignin was also observed in the case of *Pseudomonas* A514 strain [17].

The expression of glycolate oxidase, oxidase, oxidase, aldehyde oxidase, and GMC family oxidoreductase (aryl alcohol oxidase) was observed on KL–VA and these acts as an accessory enzyme and the peroxides produced by them is utilized by peroxidases for lignin degradation [27, 31]. Expression of these oxidases has also been reported recently in *Pseudomonas* A514 and *Pantoea ananatis* Sd-1 [17, 27]. The detection of NADPH:quinone oxidoreductase in *Pandoraea* strain ISTKB indicates lignin degradation by Fenton reaction. NADPH:quinone oxidoreductase overexpression on lignin and rice straw was also reported recently [17, 27, 32, 33]. Quinone oxidoreductase system is of special interest in case of lignin degradation as fungi especially brown rot used fenton chemistry for lignin degradation with the help of quinone oxidoreductase [9, 31]. The role of NADPH: quinone oxidoreductase in degradation and depolymerization of lignin is well established and reported for *Phanerochaete chrysosporium* and *Trametes versicolor* [34, 35].

Dyp-type peroxidases are fungal counterparts of peroxidase (LiP or MnP) present in bacteria for lignin degradation. The peroxidases such as DyP-type peroxidase, peroxidase, chloroperoxidase, and peroxidase-like protein were detected in *Pandoraea* sp. ISTKB genome and in proteome. Some DyPs are secreted through TAT pathway and their encapsulation has been shown to increase the enzyme’s activity [36]. There are various functions reported recently for bacterial DyPs such as depolymerization, dimer formation, and degradation of aryl ether bonds in lignin and lignin containing compounds [15, 36, 37]. Laccases can degrade lignin in the presence of mediators and there are several natural mediators observed during lignin degradation [38, 39]. Two laccase genes were discovered in the genome and found to be functionally active in this strain. Laccases are reported for ether linkage (aryl β-O-4) and β-1 bond cleavage on lignin model dimers. The degradation of phenolic as well as non-phenolic substrate in the presence of mediators by laccases has also been reported [40, 41]. Formate dehydrogenase coverts formate into carbon dioxide and these formate radicals induce MnP activity, as they can use formate as peroxide in the absence of H_2_O_2_ [31]. Formyl transferase is reported for oxalate degradation and oxalate forms complex with Mn^3+^ (MnP oxidizes Mn^2+^–Mn^3+^) and the complex acts as diffusible redox mediator for the degradation of phenolics in lignin [31]. The expression of quinone oxidoreductase, acetyl-CoA acetyltransferase, enoyl-CoA hydratase, dehydrogenase (responsible for cleavage of ether linkage), and cytochrome peroxidase was expressed on lignin, but other known bacterial lignin degrader was not observed in *Bacillus ligniniphilus* L1 expression profile [33]. The catalase/hydroperoxidase, multicopper oxidase, GMC oxidoreductase, glutathione *S*-transferase, and quinone oxidoreductases were observed in the secretome of *P. ananatis* Sd-1 on rice straw [27]. In addition to these proteins, various other proteins were also expressed in *Pandoraea* sp. strain ISTKB that are responsible for lignin degradation.

The presence of demethylases, methyltransferases, and SAM-dependent methyltransferase indicated demethylation or rearrangement of methyl group during lignin degradation [42]. Demethylation is an important process in conversion of lignin-derived aromatic intermediates into common central intermediates such as catechol, protocatechuate, or gallate that further undergo ring cleavage. Demethylation system removes methyl group from methoxy-substituted lignin-derived aromatic compounds such as syringate, vanillate, or guaiacol in the presence of cofactors. The demethylases include Rieske type ([2Fe–2S] cluster) and reductase (a flavin and a [2Fe–2S]) redox center. The demethylases or methyltransferases were also reported and functionally validated in *Pseudomonas* and *Acinetobacter* [9, 42, 43]. Several acyl-CoA synthetases, acyl-CoA hydratases/lyases, acyl-CoA transferase, acetyl-CoA-acetyl transferases, and decarboxylases have been discovered in *Pandoraea* sp. ISTKB genome and in expression profile. These enzymes help in activation and decarboxylation of aromatic compounds (hydroxycinnamates, carboxyvanillin) and play an important role in diversion of substrate towards central degradation [42–44]. The expression of both protocatechuate 3,4-dioxygenase and protocatechuate 4,5-dioxygenase on both KL–VA indicated that this strain has both functional *ortho* and *meta* cleavage pathway for degradation of lignin and its derivatives. The expression of metacleavage outperformed ortho pathway on vanillic acid. The presence of both *ortho* and *meta* cleavage pathways in single strain is rare phenomenon and the ortho cleavage pathway was found to be dominant among lignin degrading bacteria [9, 23]. The expression of both *ortho* and *meta* cleavage pathways in this strain illustrates its robust metabolic machinery for the degradation of aromatic compounds.

There are various glutathione-dependent enzymes identified in *Pandoraea* sp. ISTKB and glutathione has been known for detoxification mechanism and stress-related response. However, glutathione-dependent cleavage of β-aryl ether linkages (most dominant linkage in lignin) by β-etherase has also been described in *Novosphingobium*, *Sphingobium* SYK-6, *Novosphingobium* sp. PP1Y, and *Thiobacillus denitrificans* ATC 25259 [15, 45, 46]. Therefore, the presence of glutathione enzymes can help in lignin degradation in this strain. Superoxide dismutase and catalase–peroxidases were recently reported for lignin or lignin model compound in *Sphingobacterium* sp. T2 and *Amycolatopsis* sp. 75iv2, respectively, and these were also observed on KL–VA in this strain [47, 48].

Dehydrogenase acts on toxic aldehydes and converts them into their less toxic intermediates inside cells and also reported for cleavage of ether bond [43, 44]. There are various dehydrogenases observed in this strain and these might play important role in ether linkage degradation. The dehydrogenase-mediated degradation of ether linkage in lignin model compounds by SG61-1L and Lig DEG enzyme system in *Sphingobium* sp. SYK6 has been well documented [42, 49]. The combined action of alcohol dehydrogenase from short-chain dehydrogenase/reductase family and glutathione *S*-transferases has been show to degrade ether linkage (most prominent linkage in lignin 50–70%) in lignin model compounds [50]. The pathway for cleavage of β-aryl ether linkage in lignin by NAD-dependent dehydrogenases (LigD, LigO, and LigL) and the glutathione-dependent lyase (LigG) was structurally and biochemically characterized [51]. There are glutathione enzymes, superoxide dismutase, catalases, alkyl hydroperoxidase, thioredoxin, glyoxylase, aldo/keto reductase, and peroxiredoxin identified in *Pandoraea* sp. ISTKB. The presence of theses stress response and detoxification proteins has also been reported in genome sequence of *Pseudomonas fluorescens* Pf-5 [52]. The specificity of aldo/keto reductase against various lignin-derived phenolics, aldehyde, and fermentable inhibitors was demonstrated and was also shown to produce ROS and initiate fenton reaction [53]. Alky hydroperoxide reductase has greater catalytic efficiency under low H_2_O_2_ concentration and is responsible for the detoxification of organic hydroperoxides, as catalases cannot degrade organic hydroperoxides [54]. The analysis of such a diverse set of proteins and their level of expression helped us to identify the important enzymes responsible for lignin or aromatic compound degradation that will further provide opportunity for lignocellulosic biomass valorization.

## Conclusion

The genomic and proteomic analysis of *Pandoraea* sp. ISTKB revealed the presence of various candidate genes responsible for lignin degradation and PHA production. GO analysis of genomic and proteomic data also supported the findings. The peroxidase-accessory enzyme system, fenton reaction, and ‘CoA’-mediated degradation of phenylacetate and benzoate are the major pathways observed for lignin degradation. The gene cluster responsible for lignin degradation and PHA production was found to be functionally active. The functional analysis supported genomic findings and a strong antioxidant and stress responsive machinery for the survival and metabolism of lignin or aromatic compounds was observed. Some secondary metabolites such as lassopeptide unique to this strain were also predicted that needs to be validated. The study indicated the pathways and enzymes important for metabolism of lignin or aromatic compounds that can be applied in the future for value addition to lignocellulosics.

## Methods

The draft genome of *Pandoraea* sp. ISTKB was sequenced using the Illumina MiSeq platform, and the raw data processing, quality reads, assembly, scaffold generation, and genes prediction were carried out as described earlier [19]. Arrangement of genes of *Pandoraea* sp. strain ISTKB with respect to its genome was performed using clicO FS, i.e., circular layout interactive converter free services [55]. The proteins having signal sequence were identified using the SignalP 3.0 software [56]. The annotation and analysis of *Pandoraea* sp. ISTKB genome were also performed by Rapid Annotations using Subsystems technology (RAST). The RAST subsystem classification followed by pathway analysis was performed [57, 58]. GO analysis was performed and the genes predicted in genome have been classified into major biological processes, cellular component, and molecular functions using Blast2GO [59]. To identify the potential involvement of the genes of *Pandoraea* sp. ISTKB in biological pathways, genes were mapped to reference canonical pathways in Kyoto encyclopedia of genes’ and genomes’ (KEGG) database. The output of KEGG analysis includes KEGG orthology (KO) assignments and corresponding enzyme commission (EC) numbers and metabolic pathways of genes using KEGG automated annotation server KAAS (http://www.genome.jp/kaas-bin/kaasmain) [60]. A total of 5568 genes for *Pandoraea* sp. ISTKB were provided as input to KEGG–KAAS and genes involved in different pathways were further classified into 22 functional pathways. The antimicrobial and secondary metabolite clusters were predicted by antiSMASH 3.0 and genomic islands were predicted using islandviewer4 [61, 62].

### Culture conditions and sample preparation for proteomic analysis

*Pandoraea* sp. ISTKB was grown in mineral medium (MM) containing vanillic acid and kraft lignin as sole carbon source. The composition of MM was the same as described earlier [6]. A single colony was transferred from LB plate to broth and incubated overnight at 30 °C and 165 rpm. One milliliter of overnight culture was transferred to fresh 100 ml LB media and allowed to grow until OD_600_ reached around 0.5. The cells were pelleted, washed twice with phosphate-buffered saline (PBS), and inoculated in flask containing VA and KL having initial OD of around 0.06. Bacteria were grown at 30 °C, 165 rpm and the OD was monitored at regular interval. The culture was harvested during exponential growth phase for proteomics study. Cells were pelleted by cold centrifugation at 10,000 rpm for 15 min washed with PBS and then resuspended in lysis buffer followed by sonication as described earlier [6]. Total protein concentration was estimated by Bradford method, and then, digestion was performed taking equal volume of proteins from both KL and VA.

### Digestion of proteins, LC–MS/MS analyses, and data analysis

The protein concentration of 25 µg from both KL and VA was reduced with 5 mM concentration TCEP for 10 min at room temperature and further alkylated with 15 mM iodoacetamide in dark at room temperature for 30 min. The sample was diluted to 0.6 M final Gn-HCl concentration with 25 mM ammonium bicarbonate buffer. For digestion of protein, trypsin was added in a trypsin-to-lysate ratio of 1:50 after and incubation was performed overnight at 37 °C. The supernatant was vacuum dried and the peptides were reconstituted in 5% formic acid followed by purification using C18 silica cartridge and dried using speed vac. The dried pellets were resuspended in buffer-A (5% acetonitrile/0.15 formic acid).

The peptides were analyzed using EASY-nLC 1000 system (Thermo Fisher Scientific) coupled to QExtractive mass spectrometer (Thermo Fisher Scientific) equipped with nanoelectrospray ion source. 1 µg of peptide mixture was loaded on precolumn and resolved using 15 cm Pico Frit filled with 1.8 um C18-resin (Dr. Maeisch). The sample was run for 90 min and the peptides were eluted with a 0–40% gradient of buffer B (95% acetonitrile/0.1% formic acid) at a flow rate of 300 nl/min. the QExtractive was operated using the Top10 HCD data-dependent acquisition mode with a full-scan resolution of 70,000 at *m/z* 400. The MS/MS scans were acquired at a resolution of 17500 at *m/z* 400. Lock mass option was enabled for polydimethylcyclosiloxane (PCM) ions (*m/z* = 445.120025) for internal recalibration during the run. MS identification of Q extractive files was analyzed by the MaxQuant software and searched against databases at a false-discovery rate (FDR) of 1%. A total of protein groups were identified and were further filtered according to the label-free quantitation (LFQ) intensity values and their respective fold change values were calculated. Heat map and profile plots were against the protein groups filtered based on the normalized LFQ intensity values using the Perseus software. The proteins with at least two unique peptides detected were selected for quantification and differential expression study.

## Additional files

**Additional file 1: Figure S1.** Representation of various proteins responsible for respiratory machinery of *Pandoraea* sp. ISTKB. **Figure S2.** Transcriptional regulators identified in the genome of *Pandoraea* sp. ISTKB. **Figure S3.** Representation of various transporters present in the genome of *Pandoraea* sp. ISTKB. **Figure S4.** Representation of major oxidoreductases responsible for lignin and aromatic compound degradation. **Figure S5.** Representation of proteins into various groups involved in stress regulation mechanism. **Figure S6.** Representation of genomic islands predicted by Islandviewer 4 in the *Pandoraea* sp. ISTKB genome. **Table S1.** KEGG Pathway classification of *Pandoraea* sp. ISTKB. **Table S2.** Annotation of Monooxygenase genes identified in the genome of *Pandoraea* sp. ISTKB. **Table S3.** Annotation of Dioxygenase genes identified in the genome of *Pandoraea*sp. ISTKB. **Table S4.** Annotation of Peroxidase genes identified in the genome of *Pandoraea* sp. ISTKB. **Table S5.** Peripheral pathways for catabolism of aromatic compounds. **Table S6.** Annotation of genes responsible for metabolism of central aromatic intermediates. **Table S7.** Annotation of genes related to Glutathione metabolism and stress response. **Table S8.** Represents secondary metabolite clusters identified in *Pandoraea* sp. ISTKB genome.

**Additional file 2: Table S9.** Detail of genomic islands identified in *Pandoraea* sp. ISTKB genome.

**Additional file 3: Table S10.** Other differentially expressed proteins (related to PHA metabolism, dehydrogenase, reductases, transferases, esterases and hydrolases) on kraft lignin.

## References

[1] Coenye T, Falsen E, Hoste B, Ohlén M, Goris J, Govan J, Gillis M, Vandamme P (2000). NEW TAXA-proteobacteria-description of *Pandoraea* gen. nov. with *Pandoraea api*sta sp. nov., *Pandoraea pulmonicola* sp. nov., *Pandoraea pnomenusa* sp. nov., *Pandoraea sputorum* sp. nov. and *Pandoraea*. Int J Syst Evol Microbiol.

[2] Daneshvar MI, Hollis DG, Steigerwalt AG, Whitney AM, Spangler L, Douglas MP, Jordan JG, MacGregor JP, Hill BC, Tenover FC (2001). Assignment of CDC weak oxidizer group 2 (WO-2) to the genus *Pandoraea* and characterization of three new *Pandoraea* genomospecies. J Clin Microbiol.

[3] Anandham R, Indiragandhi P, Kwon SW, Sa TM, Jeon CO, Kim YK, Jee HJ (2010). *Pandoraea thiooxydans* sp. nov., a facultatively chemolithotrophic, thiosulfate-oxidizing bacterium isolated from rhizosphere soils of sesame (*Sesamum indicum* L.). Int J Syst Evol Microbiol.

[4] Sahin N, Tani A, Kotan R, Sedláček I, Kimbara K, Tamer AU (2011). *Pandoraea oxalativorans* sp. nov., *Pandoraea faecigallinarum* sp. nov. and *Pandoraea vervacti* sp. nov., isolated from oxalate-enriched culture. Int J Syst Evol Microbiol.

[5] Chen CY, Kuo JT, Cheng CY, Huang YT, Ho IH, Chung YC (2009). Biological decolorization of dye solution containing malachite green by *Pandoraea pulmonicola* YC32 using a batch and continuous system. J Hazard Mater.

[6] Kumar M, Singh J, Singh MK, Singhal A, Thakur IS (2015). Investigating the degradation process of kraft lignin by β-proteobacterium, *Pandoraea* sp. ISTKB. Environ Sci Pollut Res.

[7] Tian JH, Pourcher AM, Bouchez T, Gelhaye E, Peu P (2014). Occurrence of lignin degradation genotypes and phenotypes among prokaryotes. Appl Microbiol Biotechnol.

[8] Priyadarshinee R, Kumar A, Mandal T, Dasguptamandal D (2016). Unleashing the potential of ligninolytic bacterial contributions towards pulp and paper industry: key challenges and new insights. Environ Sci Pollut Res.

[9] Bugg TD, Ahmad M, Hardiman EM, Rahmanpour R (2011). Pathways for degradation of lignin in bacteria and fungi. Nat Prod Rep.

[10] Brown ME, Chang MC (2014). Exploring bacterial lignin degradation. Curr Opin Chem Biol.

[11] Beckham GT, Johnson CW, Karp EM, Salvachúa D, Vardon DR (2016). Opportunities and challenges in biological lignin valorization. Curr Opin Biotechnol.

[12] Baldrian P, López-Mondéjar R (2014). Microbial genomics, transcriptomics and proteomics: new discoveries in decomposition research using complementary methods. Appl Microbiol Biotechnol.

[13] Cragg SM, Beckham GT, Bruce NC, Bugg TD, Distel DL, Dupree P, Etxabe AG, Goodell BS, Jellison J, McGeehan JE (2015). Lignocellulose degradation mechanisms across the Tree of Life. Curr Opin Chem Biol.

[14] Qin W (2016). Recent developments in using advanced sequencing technologies for the genomic studies of lignin and cellulose degrading microorganisms. Int J Biol Sci.

[15] de Gonzalo G, Colpa DI, Habib MH, Fraaije MW (2016). Bacterial enzymes involved in lignin degradation. J Biotechnol.

[16] Manavalan A, Adav SS, Sze SK (2011). iTRAQ-based quantitative secretome analysis of *Phanerochaete chrysosporium*. J Proteomics.

[17] Lin L, Cheng Y, Pu Y, Sun S, Li X, Jin M, Pierson EA, Gross DC, Dale BE, Dai SY (2016). Systems biology-guided biodesign of consolidated lignin conversion. Green Chem.

[18] Singh MK, Kumar M, Thakur IS (2017). Proteomic characterization and schizophyllan production by Schizophyllum commune ISTL04 cultured on *Leucaena leucocephala* wood under submerged fermentation. Bioresour Technol.

[19] Kumar M, Gazara RK, Verma S, Kumar M, Verma PK, Thakur IS (2016). Genome sequence of *Pandoraea* sp. ISTKB, a lignin-degrading betaproteobacterium, isolated from rhizospheric soil. Genome Announc.

[20] Kumar M, Singhal A, Verma PK, Thakur IS (2017). Production and characterization of polyhydroxyalkanoate from lignin derivatives by *Pandoraea* sp. ISTKB. ACS Omega.

[21] Varman AM, He L, Follenfant R, Wu W, Wemmer S, Wrobel SA, Tang YJ, Singh S (2016). Decoding how a soil bacterium extracts building blocks and metabolic energy from ligninolysis provides road map for lignin valorization. Proc Natl Acad Sci.

[22] Kumar M, Singhal A, Thakur IS (2016). Comparison of submerged and solid state pretreatment of sugarcane bagasse by *Pandoraea* sp. ISTKB: enzymatic and structural analysis. Bioresour Technol.

[23] Fuchs G, Boll M, Heider J (2011). Microbial degradation of aromatic compounds—from one strategy to four. Nat Rev Microbiol.

[24] Cabiscol Català E, Tamarit Sumalla J, Ros Salvador J (2000). Oxidative stress in bacteria and protein damage by reactive oxygen species. Int Microbiol.

[25] Hasanuzzaman M, Nahar K, Hossain MS, Mahmud JA, Rahman A, Inafuku M, Oku H, Fujita M (2017). Coordinated actions of glyoxalase and antioxidant defense systems in conferring abiotic stress tolerance in plants. Int J Mol Sci.

[26] Rehm BH (2010). Bacterial polymers: biosynthesis, modifications and applications. Nat Rev Microbiol.

[27] Ma J, Zhang K, Liao H, Hector SB, Shi X, Li J, Liu B, Xu T, Tong C, Liu X (2016). Genomic and secretomic insight into lignocellulolytic system of an endophytic bacterium Pantoea ananatis Sd-1. Biotechnol Biofuels.

[28] Garrido-Pertierra A, Cooper RA (1981). Identification and purification of distinct isomerase and decarboxylase enzymes involved in the 4-hydroxyphenylacetate catabolic pathway of *Escherichia coli*. FEBS J.

[29] Rather LJ, Knapp B, Haehnel W, Fuchs G (2010). Coenzyme A-dependent aerobic metabolism of benzoate via epoxide formation. J Biol Chem.

[30] Teufel R, Mascaraque V, Ismail W, Voss M, Perera J, Eisenreich W, Haehnel W, Fuchs G (2010). Bacterial phenylalanine and phenylacetate catabolic pathway revealed. Proc Natl Acad Sci.

[31] Dashtban M, Schraft H, Syed TA, Qin W (2010). Fungal biodegradation and enzymatic modification of lignin. Int J Biochem Mol Biol.

[32] Baldrian P, Valášková V (2008). Degradation of cellulose by basidiomycetous fungi. FEMS Microbiol Rev.

[33] Zhu D, Zhang P, Xie C, Zhang W, Sun J, Qian W-J, Yang B (2017). Biodegradation of alkaline lignin by *Bacillus ligniniphilus* L1. Biotechnol Biofuels.

[34] Constam D, Muheim A, Zimmermann W, Fiechter A (1991). Purification and partial characterization of an intracellular NADH: quinone oxidoreductase from *Phanerochaete chrysosporium*. Microbiology.

[35] Lee S, Moon D, Choi HT, Song H (2007). Purification and characterization of an intracellular NADH: quinone reductase from *Trametes versicolor*. J Microbiol.

[36] Rahmanpour R, Rea D, Jamshidi S, Fülöp V, Bugg TD (2016). Structure of *Thermobifida fusca* DyP-type peroxidase and activity towards kraft lignin and lignin model compounds. Arch Biochem Biophys.

[37] Singh R, Grigg JC, Qin W, Kadla JF, Murphy ME, Eltis LD (2013). Improved manganese-oxidizing activity of DypB, a peroxidase from a lignolytic bacterium. ACS Chem Biol.

[38] Christopher LP, Yao B, Ji Y (2014). Lignin biodegradation with laccase-mediator systems. Front Energy Res.

[39] Munk L, Sitarz AK, Kalyani DC, Mikkelsen JD, Meyer AS (2015). Can laccases catalyze bond cleavage in lignin?. Biotechnol Adv.

[40] Sondhi S, Sharma P, George N, Chauhan PS, Puri N, Gupta N (2015). An extracellular thermo-alkali-stable laccase from *Bacillus tequilensis* SN4, with a potential to biobleach softwood pulp. 3 Biotech.

[41] Shi X, Liu Q, Ma J, Liao H, Xiong X, Zhang K, Wang T, Liu X, Xu T, Yuan S (2015). An acid-stable bacterial laccase identified from the endophyte Pantoea ananatis Sd-1 genome exhibiting lignin degradation and dye decolorization abilities. Biotechnol Lett.

[42] Masai E, Katayama Y, Fukuda M (2007). Genetic and biochemical investigations on bacterial catabolic pathways for lignin-derived aromatic compounds. Biosci Biotechnol Biochem.

[43] Abdelaziz OY, Brink DP, Prothmann J, Ravi K, Sun M, García-Hidalgo J, Sandahl M, Hulteberg CP, Turner C, Lidén G (2016). Biological valorization of low molecular weight lignin. Biotechnol Adv.

[44] Pérez-Pantoja D, González B, Pieper D, Timmis KN (2010). Aerobic degradation of aromatic hydrocarbons. Handbook of hydrocarbon and lipid microbiology.

[45] Tanamura K, Abe T, Kamimura N, Kasai D, Hishiyama S, Otsuka Y, Nakamura M, Kajita S, Katayama Y, Fukuda M (2011). Characterization of the third glutathione *S*-transferase gene involved in enantioselective cleavage of the β-aryl ether by *Sphingobium* sp. strain SYK-6. Biosci Biotechnol Biochem.

[46] Picart P, Müller C, Mottweiler J, Wiermans L, Bolm C, Domínguez de María P, Schallmey A (2014). From gene towards selective biomass valorization: bacterial β-etherases with catalytic activity on lignin-like polymers. Chemsuschem.

[47] Brown ME, Walker MC, Nakashige TG, Iavarone AT, Chang MC (2011). Discovery and characterization of heme enzymes from unsequenced bacteria: application to microbial lignin degradation. J Am Chem Soc.

[48] Rashid GM, Taylor CR, Liu Y, Zhang X, Rea D, Fülöp V, Bugg TD (2015). Identification of manganese superoxide dismutase from *Sphingobacterium* sp. T2 as a novel bacterial enzyme for lignin oxidation. ACS Chem Biol.

[49] Palamuru S, Dellas N, Pearce SL, Warden AC, Oakeshott JG, Pandey G (2015). Phylogenetic and kinetic characterization of a suite of dehydrogenases from a newly isolated bacterium, strain SG61-1L, that catalyze the turnover of guaiacylglycerol-β-guaiacyl ether stereoisomers. Appl Environ Microbiol.

[50] Sato Y, Moriuchi H, Hishiyama S, Otsuka Y, Oshima K, Kasai D, Nakamura M, Ohara S, Katayama Y, Fukuda M (2009). Identification of three alcohol dehydrogenase genes involved in the stereospecific catabolism of arylglycerol-β-aryl ether by *Sphingobium* sp. strain SYK-6. Appl Environ Microbiol.

[51] Pereira JH, Heins RA, Gall DL, McAndrew RP, Deng K, Holland KC, Donohue TJ, Noguera DR, Simmons BA, Sale KL (2016). Structural and biochemical characterization of the early and late enzymes in the lignin β-aryl ether cleavage pathway from *Sphingobium* sp. SYK-6. J Biol Chem.

[52] Paulsen IT, Press CM, Ravel J, Kobayashi DY, Myers GS, Mavrodi DV, DeBoy RT, Seshadri R, Ren Q, Madupu R (2005). Complete genome sequence of the plant commensal *Pseudomonas fluorescens* Pf-5. Nat Biotechnol.

[53] Tramontina R, Cairo JPLF, Liberato MV, Mandelli F, Sousa A, Santos S, Rabelo SC, Campos B, Ienczak J, Ruller R (2017). The *Coptotermes gestroi* aldo–keto reductase: a multipurpose enzyme for biorefinery applications. Biotechnol Biofuels.

[54] Seaver LC, Imlay JA (2001). Alkyl hydroperoxide reductase is the primary scavenger of endogenous hydrogen peroxide in *Escherichia coli*. J Bacteriol.

[55] Cheong WH, Tan YC, Yap SJ, Ng KP (2015). ClicO FS: an interactive web-based service of Circos. Bioinformatics.

[56] Bendtsen JD, Nielsen H, von Heijne G, Brunak S (2004). Improved prediction of signal peptides: SignalP 3.0. J Mol Biol.

[57] Aziz RK, Bartels D, Best AA, DeJongh M, Disz T, Edwards RA, Formsma K, Gerdes S, Glass EM, Kubal M (2008). The RAST server: rapid annotations using subsystems technology. BMC Genomics.

[58] Overbeek R, Olson R, Pusch GD, Olsen GJ, Davis JJ, Disz T, Edwards RA, Gerdes S, Parrello B, Shukla M (2013). The SEED and the rapid annotation of microbial genomes using subsystems technology (RAST). Nucleic Acids Res.

[59] Conesa A, Götz S, García-Gómez JM, Terol J, Talón M, Robles M (2005). Blast2GO: a universal tool for annotation, visualization and analysis in functional genomics research. Bioinformatics.

[60] Moriya Y, Itoh M, Okuda S, Yoshizawa AC, Kanehisa M (2007). KAAS: an automatic genome annotation and pathway reconstruction server. Nucleic Acids Res.

[61] Weber T, Blin K, Duddela S, Krug D, Kim HU, Bruccoleri R, Lee SY, Fischbach MA, Müller R, Wohlleben W (2015). antiSMASH 3.0—a comprehensive resource for the genome mining of biosynthetic gene clusters. Nucleic Acids Res.

[62] Bertelli C, Laird MR, Williams KP, Lau BY, Hoad G, Winsor GL, Brinkman FS, Group SFURC (2017). IslandViewer 4: expanded prediction of genomic islands for larger-scale datasets. Nucleic Acids Res.

