# Supplementary material for: Genomic and proteomic analysis of lignin degrading and polyhydroxyalkanoate accumulating β-proteobacterium Pandoraea sp. ISTKB
Source: Biotechnol Biofuels. 2018 Jun 5;11:154. doi: 10.1186/s13068-018-1148-2 (PMC5987411; doi:10.1186/s13068-018-1148-2)
Supplement: Supplementary file 1 — Additional file 1: Figure S1. Representation of various proteins responsible for respiratory machinery of Pandoraea sp. ISTKB. Figure S2. Transcriptional regulators identified in the genome of Pandoraea sp. ISTKB. Figure S3. Representation of various transporters present in the genome of Pandoraea sp. ISTKB. Figure S4. Representation of major oxidoreductases responsible for lignin and aromatic compound degradation. Figure S5. Representation of proteins into various groups involved in stress regulation mechanism. Figure S6. Representation of genomic islands predicted by Islandviewer 4 in the Pandoraea sp. ISTKB genome. Table S1. KEGG Pathway classification of Pandoraea sp. ISTKB. Table S2. Annotation of Monooxygenase genes identified in the genome of Pandoraea sp. ISTKB. Table S3. Annotation of Dioxygenase genes identified in the genome of Pandoraeasp. ISTKB. Table S4. Annotation of Peroxidase genes identified in the genome of Pandoraea sp. ISTKB. Table S5. Peripheral pathways for catabolism of aromatic compounds. Table S6. Annotation of genes responsible for metabolism of central aromatic intermediates. Table S7. Annotation of genes related to Glutathione metabolism and stress response. Table S8. Represents secondary metabolite clusters identified in Pandoraea sp. ISTKB genome. [file 13068_2018_1148_MOESM1_ESM.pdf]

Additional file 1

Title: Genomic and proteomic analysis of lignin degrading and polyhydroxyalkanoate accumulating β-proteobacterium *Pandoraea* sp. ISTKB

Author names and affiliations:

Madan Kumar[a], Sandhya Verma [b], Rajesh Kumar Gazara [b], Manish Kumar[a], Ashok Pandey[c], Praveen Kumar Verma[b]* and Indu Shekhar Thakur[a]*

[a]*School of Environmental Sciences, Jawaharlal Nehru University, New Delhi- 110067, India*

[b]*National Institute of Plant Genome Research, Aruna Asaf Ali Marg, New Delhi-110067, India*

[c]*CSIR-Indian Institute of Toxicology Research, 31 MG Marg, Lucknow-226 001, India*

*Corresponding Author

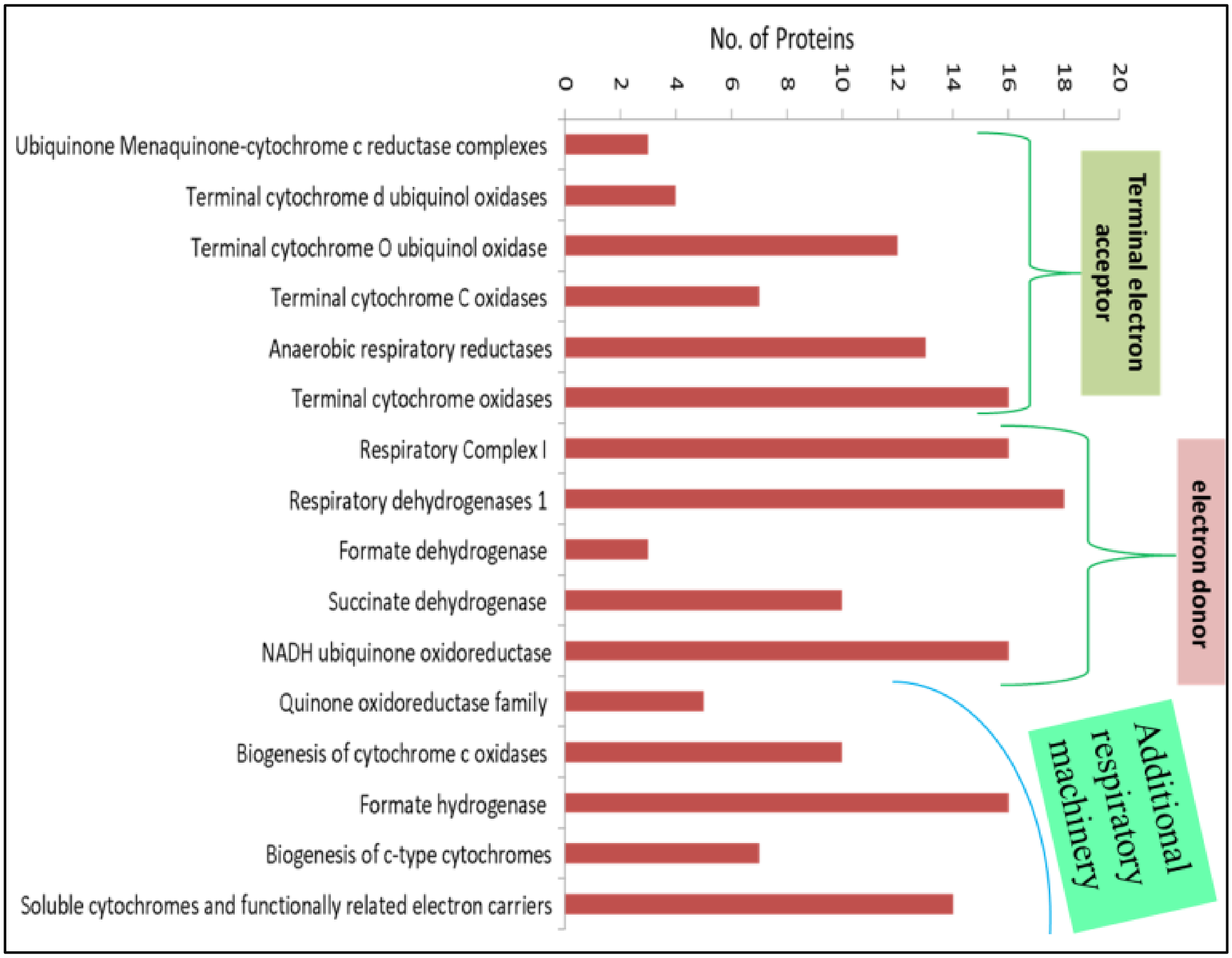

Fig. S1. Representation of various proteins responsible for respiratory machinery of *Pandoraea*sp. ISTKB.

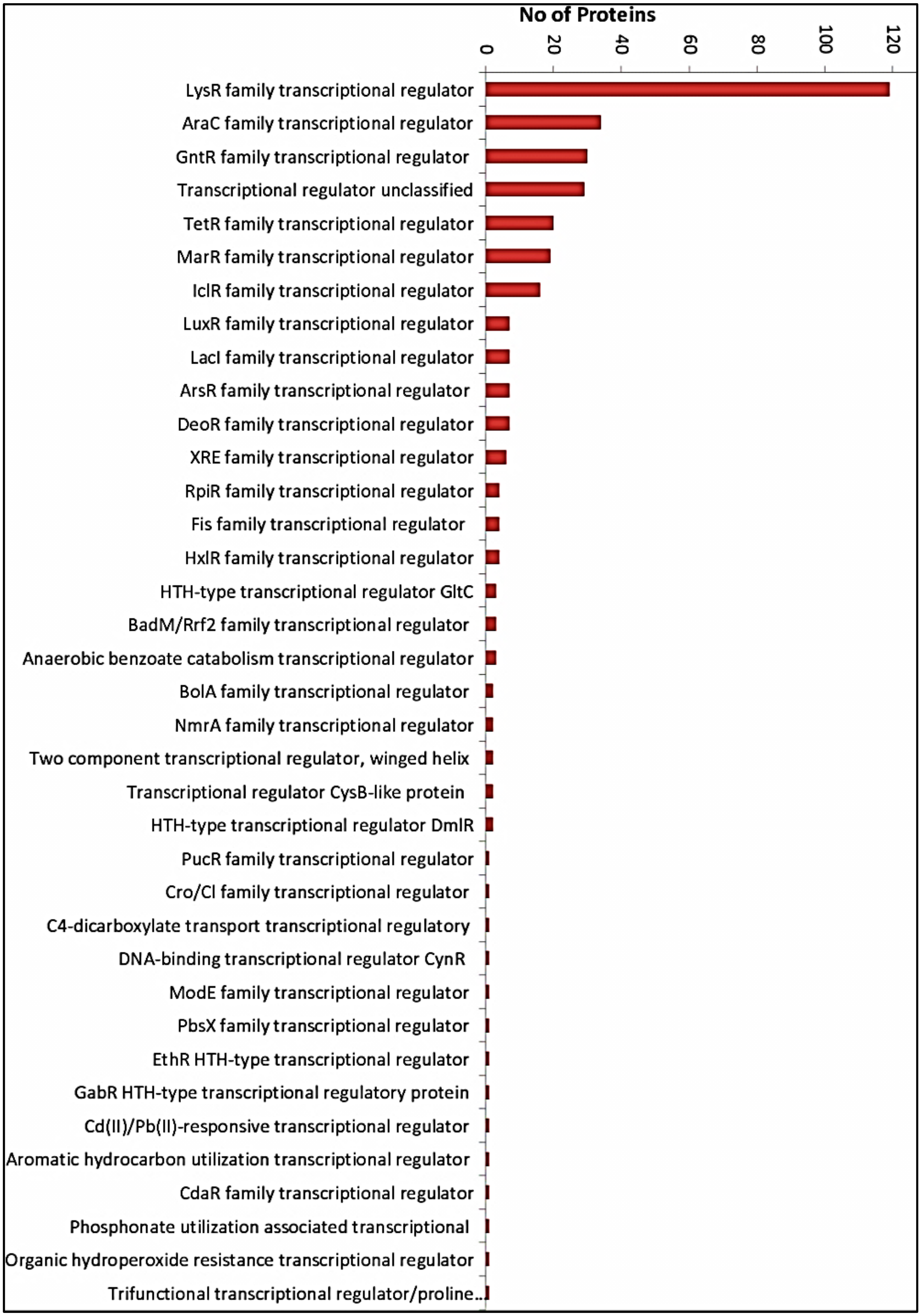

Fig. S2. Transcriptional regulators identified in the genome of *Pandoraea* sp. ISTKB.

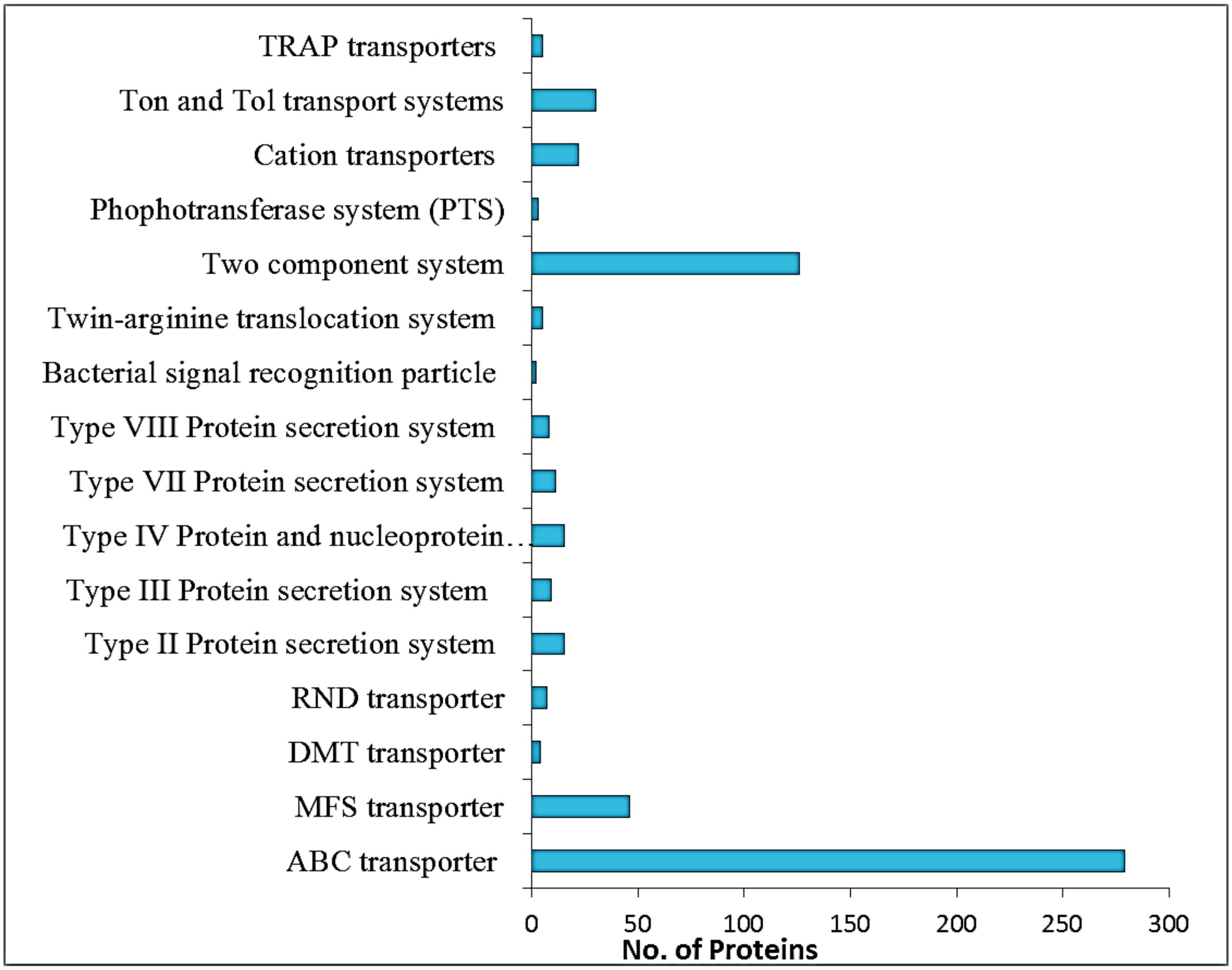

Fig. S3. Representation of various transporters present in the genome of *Pandoraea* sp. ISTKB.

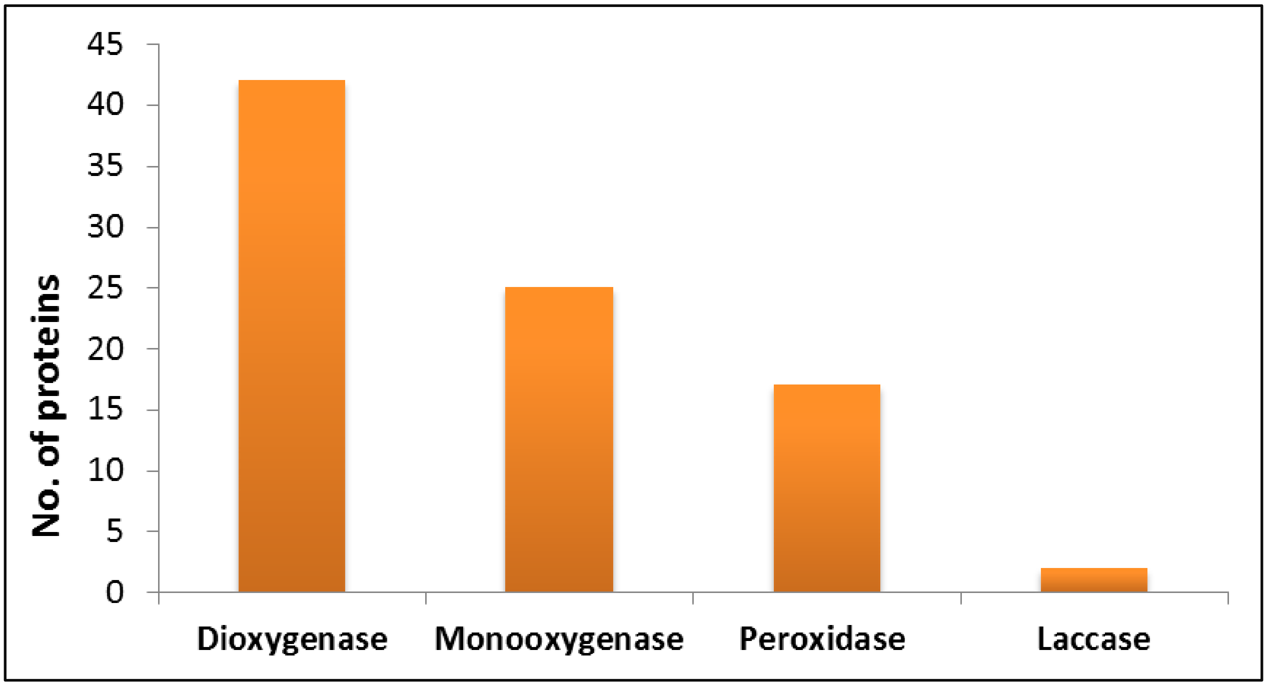

Fig. S4. Representation of major oxidoreductases responsible for lignin and aromatic compound degradation.

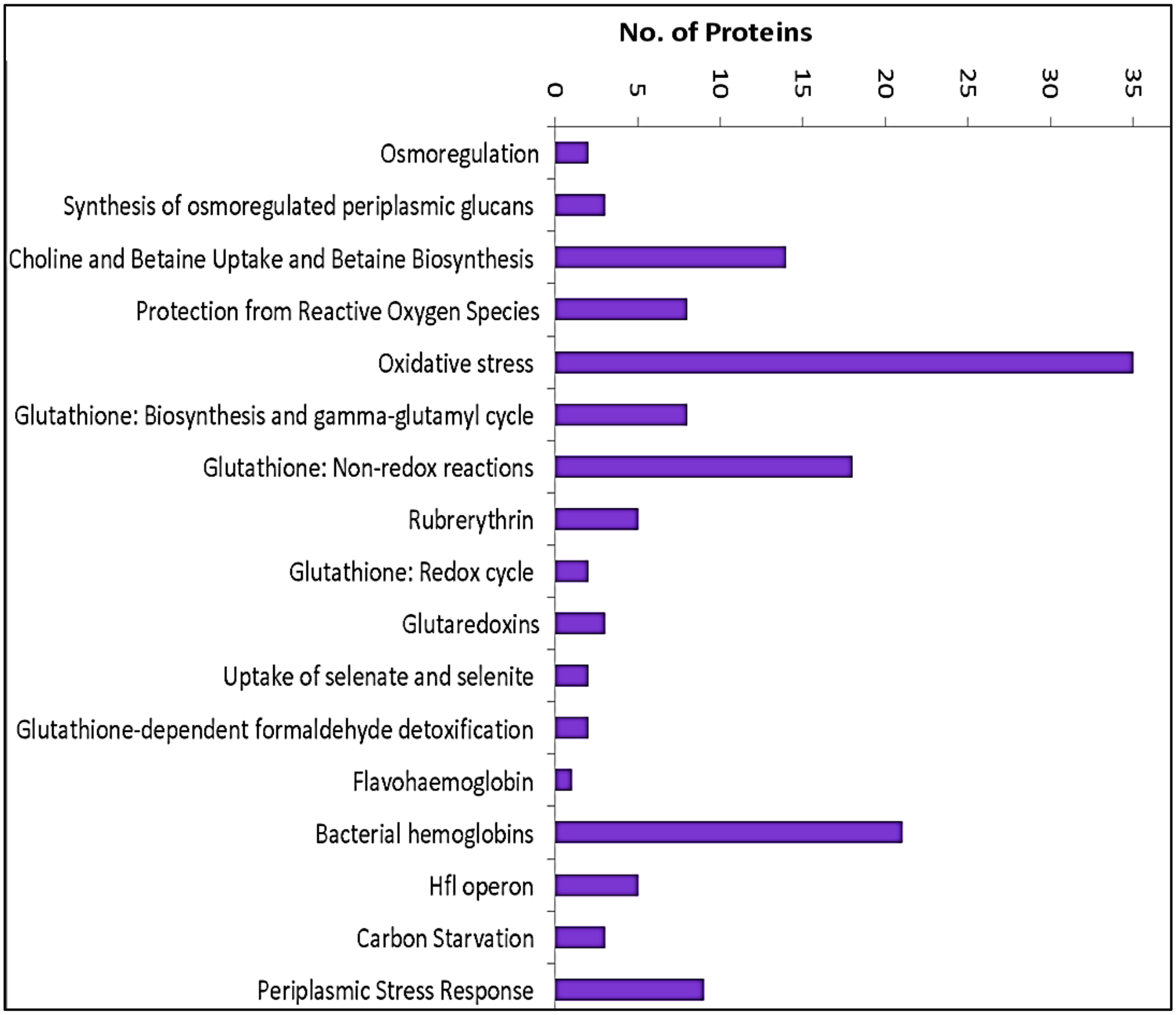

Fig. S5. Representation of proteins into various groups involved in stress regulation mechanism.

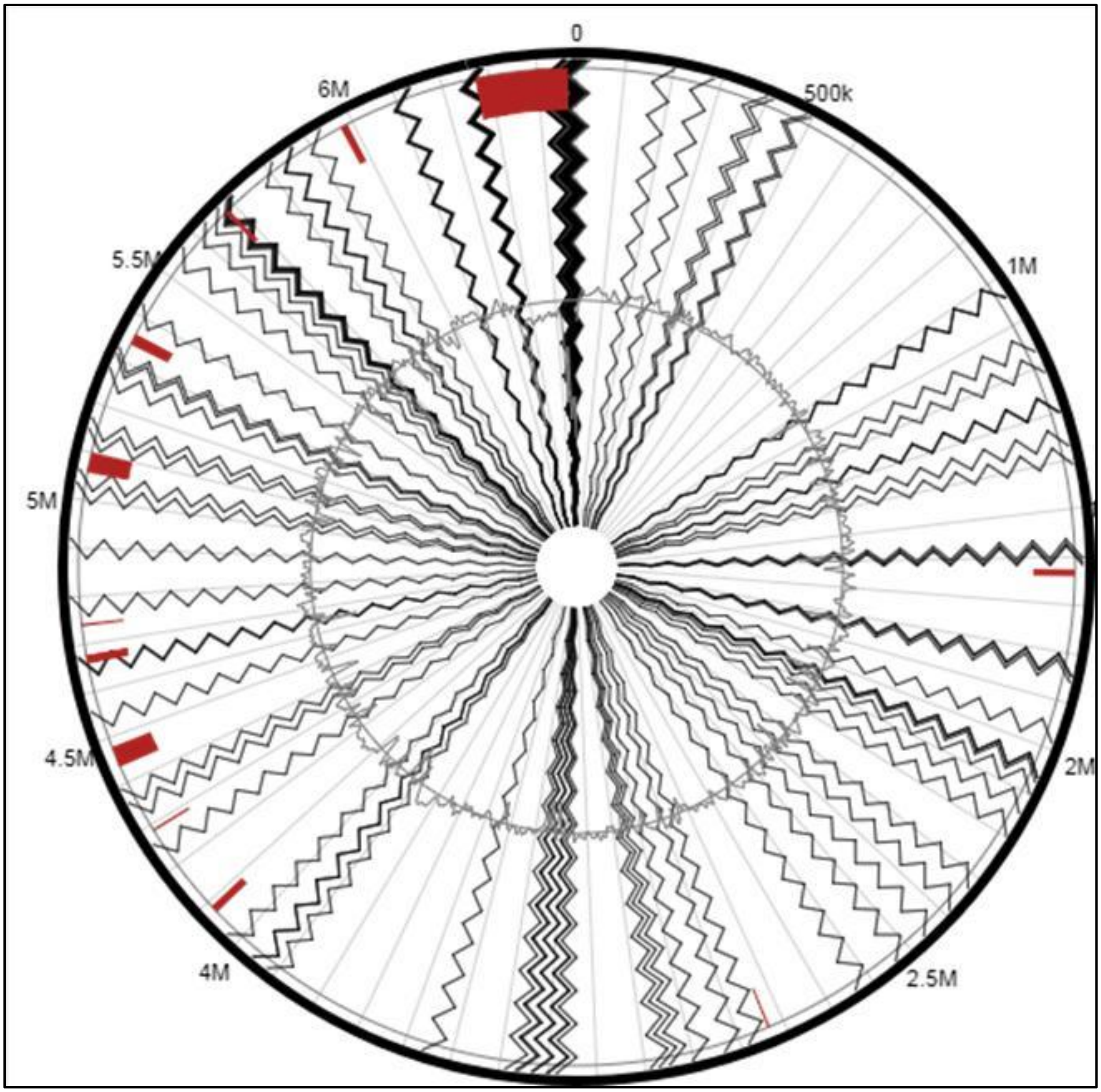

Fig. S6. Representation of genomic islands predicted by Islandviewer 4 in the *Pandoraea* sp. ISTKB genome.

Table S1 KEGG Pathway classification of *Pandoraea* sp. ISTKB.

KEGG Pathway	Number of Proteins
**Metabolism**	
Overview	286
Carbohydrate metabolism	259
Energy metabolism	179
Lipid metabolism	88
Nucleotide metabolism	106
Amino acid metabolism	318
Metabolism of other amino acids	88
Glycan biosynthesis and metabolism	45
Metabolism of cofactors and vitamins	197
Metabolism of terpenoids and polyketides	44
Biosynthesis of other secondary metabolites	41
Xenobiotics biodegradation and metabolism	116
**Genetic Information Processing**	
Transcription	4
Translation	85
Folding, sorting and degradation	42
Replication and repair	50
**Environmental Information Processing**	
Membrane transport	226
Signal transduction	146
**Cellular Processes**	
Transport and catabolism	15
Cell growth and death	22
Cellular community - prokaryotes	173
Cell motility	60

Table S2. Annotation of Monooxygenase genes identified in the genome of *Pandoraea* sp. ISTKB

Pando_Id (query)Locus tag	Pando_Id (query)Locus tag	Description	% Identity	Alignment length	E-value	Query cover
A9762_26360	AHB77617.1	FAD-containing monooxygenase EthA [Pandoraea pnomenusa]	83.168	505	0	95
A9762_15430	ODS71808.1	monooxygenase [Bordetella sp. SCN 67-23]	62.532	387	2.28E-161	94
A9762_05700	SAL83236.1	monooxygenase [Burkholderia arvi]	82.522	452	0	99
A9762_00610	SAK77773.1	monooxygenase [Burkholderia ptereochthonis]	72.018	436	0	98
A9762_00860	CFB62521.1	Nitrilotriacetate monooxygenase component A [Pandoraea apista]	94.678	451	0	94
A9762_00640	EWS66112.1	Nitronate monooxygenase [Hydrogenophaga sp. T4]	64.537	313	2.2E-134	99
A9762_13280	EON12850.1	phenylalanine 4-monooxygenase [Pandoraea sp. SD6-2]	93.95	281	0	99
A9762_17475	SAL13664.1	4-hydroxybenzoate 3-monooxygenase [Paraburkholderia telluris]	67.263	391	0	99
A9762_05250	AOL06528.1	6-hydroxynicotinate 3-monooxygenase [Burkholderia lata]	84.555	382	0	98
A9762_09625	SAK76029.1	alkanesulfonate monooxygenase [Burkholderia arationis]	73.368	383	0	99
A9762_22815	OED18971.1	alkanesulfonate monooxygenase [Burkholderia sp. A2]	82.808	349	0	95
A9762_08385	SCB16369.1	anthraniloyl-CoA monooxygenase [Cupriavidus alkaliphilus]	79.821	783	0	99
A9762_04935	ODV40636.1	antibiotic biosynthesis monooxygenase [Cupriavidus sp. UYMMa02A]	64.583	96	5.31E-42	96
A9762_26815	OAK66449.1	antibiotic biosynthesis monooxygenase [Variovorax paradoxus]	71.154	104	2.12E-50	94
A9762_15430	EOM77545.1	aromaticringmonooxygenase[RhodococcusrhodniiLMG5362]	56.576	403	9.97E-140	97
A9762_20610	KYF55981.1	FAD-bindingmonooxygenase[Sorangiumcellulosum]	41.823	373	2.25E-92	93
A9762_08590	KYF90066.1	FAD-bindingmonooxygenase[Sorangiumcellulosum]	49.102	501	9.3E-145	98
A9762_11070	EHP37661.1	FAD-dependentmonooxygenase[CupriavidusbasilensisOR16]	42.13	432	3.45E-90	96
A9762_21985	SAL54713.1	flavin-containingmonooxygenaseFMO[Burkholderiaarvi]	81.604	424	0	99
A9762_00395	CUJ47974.1	Flavin-dependentmonooxygenase[Achromobactersp.2789STDY5608625]	83.03	165	6.29E-98	99
A9762_05695	ADD78448.1	4-hydroxyphenylacetate3-monooxygenase,[PantoeaananatisPA13]	52.532	158	3.72E-55	92
A9762_16310	SAL10828.1	luciferase-likemonooxygenase[Paraburkholderiahumi]	75.224	335	0	95
A9762_23090	AEI80663.1	nitronatemonooxygenase[CupriavidusnecatorN-1]	66.116	363	7.5E-151	97
A9762_23095	AEI80663.1	nitronatemonooxygenase[CupriavidusnecatorN-1]	70.137	365	5.33E-165	97
A9762_10935	AAZ62959.1	Salicylate1-monooxygenase[RalstoniaeutrophaJMP134]	70.278	360	6.2E-180	93

Table S3. Annotation of Dioxygenase genes identified in the genome of *Pandoraea*sp. ISTKB

Pando_Id (query)Locus tag	NR_Db_Id (Subject)	Description	% Identity	alignment length	E- value	Query cover
A9762_05750	ODS74090.1	2,4-dichlorophenoxyacetate dioxygenase [Bordetella sp. SCN 67-23]	64.789	284	1.78E-128	93
A9762_05265	SAL59031.1	2,5-dihydroxypyridine 5,6-dioxygenase [Burkholderiaarvi]	86.589	343	0	99
A9762_20395	SCB09348.1	2,5-dihydroxypyridine 5,6-dioxygenase [Cupriavidusalkaliphilus]	70.37	351	6.83E-169	98
A9762_23090	EON11665.1	2-nitropropane dioxygenase [Pandoraea sp. SD6-2]	91.329	346	0	93
A9762_00970	ODV41070.1	4-hydroxyphenylpyruvate dioxygenase [Cupriavidus sp. UYMMa02A]	91.317	357	0	99
A9762_07755	AFT86541.1	benzoyl-CoA 2,3-dioxygenase component A [Paraburkholderiaphenoliruptrix BR3459a]	75.708	424	0	99
A9762_22825	AJP59870.1	cysteine dioxygenase [Pandoraeavervacti]	95.61	205	5.23E-143	99
A9762_27320	AJP58034.1	dioxygenase [Pandoraeavervacti]	98.12	266	0	99
A9762_27700	AJP58034.1	dioxygenase [Pandoraeavervacti]	98.12	266	0	99
A9762_24855	ODS79332.1	extradiol ring-cleavage dioxygenase [Acidovorax sp. SCN 65-28]	72.222	288	2.28E-159	99
A9762_01340	SCB18330.1	gentisate 1,2-dioxygenase [Cupriavidusalkaliphilus]	84.77	348	0	99
A9762_15850	SAK61630.1	glyoxalase/bleomycin resistance protein/dioxygenase [Burkholderiaptereochthonis]	86.232	138	2.77E-86	99
A9762_01405	EUC18785.1	Glyoxalase/bleomycin resistance protein/dioxygenase [Burkholderia sp. BT03]	74.126	286	1.76E-159	99
A9762_12845	SAL18551.1	glyoxalase/bleomycin resistance protein/dioxygenase [Paraburkholderiaterrestris]	72.263	137	3.3E-69	97
A9762_21815	SAL80458.1	homogentisate 1,2-dioxygenase [Paraburkholderiatelluris]	82.944	428	0	97
A9762_05645	BAM71411.1	hydroxyquinol 1,2-dioxygenase [Ralstoniapickettii]	72.263	274	4.7E-144	94
A9762_16335	ODV41789.1	naphthalene 1,2-dioxygenase [Cupriavidus sp. UYMMa02A]	70.192	104	4.96E-53	99
A9762_16350	ODV10699.1	naphthalene 1,2-dioxygenase [Rubrivivax sp. SCN 70-15]	57.102	352	6.83E-139	99
A9762_07980	SCB33007.1	nitric oxide dioxygenase [Cupriavidusalkaliphilus]	60.345	406	5.04E-156	99
A9762_11040	ODS75686.1	phthalate 4,5-dioxygenase [Bordetella sp. SCN 67-23]]	64.096	415	0	93
A9762_21285	ODS69252.1	protocatechuate 3,4-dioxygenase [Bordetella sp. SCN 67-23]	71.429	119	5.36E-57	99
A9762_17445	OED18908.1	protocatechuate 3,4-dioxygenase subunit beta [Burkholderia sp. A2]	74.569	232	2.71E-126	98
A9762_21280	EGD14970.1	protocatechuate 4,5-dioxygenase beta subunit [Xanthomonasperforans 91-118]	82.394	284	4.95E-175	99
A9762_21975	SAL54674.1	putative dioxygenase [Burkholderiaarvi]	85.106	376	0	98
A9762_22350	KPD18713.1	quercetin 2,3-dioxygenase [Burkholderia sp. ST111]	76.987	239	2.72E-134	99

Contd…

Pando_Id (query)Locus tag	NR_Db_Id (Subject)	Description	% Identity	alignment length	E- value	Query cover
A9762_00640	ETF03411.1	2-nitropropanedioxygenase[AdvenellakashmirensisW13003]	63.141	312	3.29E-118	99
A9762_23095	SAL82943.1	2-nitropropanedioxygenase[Burkholderiaarvi]	64.305	367	2.58E-145	97
A9762_27235	KWW35604.1	Alpha-ketoglutarate-dependentdioxygenaseAlkB[Cupriavidusmetallidurans]	60.36	222	5.4E-82	99
A9762_01405	SAK81886.1	biphenyl2,3-dioxygenase[Burkholderiacatudaia]	66.899	287	3.17E-139	99
A9762_19835	SCB27202.1	catechol1,2-dioxygenase[Cupriavidusalkaliphilus]	69.258	283	4.86E-139	97
A9762_24855	KUJ67147.1	catechol1,2-dioxygenase[Streptomycesalbussubsp.albus]	41.958	286	3.29E-62	99
A9762_19185	GAQ27037.1	catechol2,3-dioxygenase[Ralstoniasp.NT80]	78.652	178	1.81E-102	96
A9762_19755	SAL51551.1	Chlorohydroquinone/hydroquinone1,2-dioxygenase[Burkholderiaturbans]	43.533	317	1.35E-84	99
A9762_17315	EPX94702.1	dioxygenase[Ralstoniasp.AU12-08]	84.585	253	8.69E-160	91
A9762_17955	ODV19431.1	dioxygenase[Rhodanobactersp.SCN68-63]	53.03	264	8.51E-91	96
A9762_14645	SAK54841.1	glyoxalase/bleomycinresistanceprotein/dioxygenase[Burkholderiaglebae]	73.188	138	4.31E-72	99
A9762_19155	EKF18187.1	Glyoxalase/bleomycinresistanceprotein/dioxygenase[Nitratireductorpacificuspht-3B]	51.128	133	8.5E-38	85
A9762_12845	SAL69811.1	glyoxalase/bleomycinresistanceprotein/dioxygenase[Paraburkholderiatelluris]	69.343	137	5.46E-67	97
A9762_11010	SCK52185.1	Phenylpropionatedioxygenase,largeterminalsubunit[Variovoraxsp.HW608]	73.193	332	0	96
A9762_25450	CCA87771.1	putativeglyoxalase/bleomycinresistanceprotein/dioxygenase(phnB)[RalstoniasyzygiiR24]	58.904	146	3.01E-51	97
A9762_19755	AAK26513.1	putativehydroquinonemetacleavagedioxygenase(plasmid)[Pseudomonasputida]	78.481	316	0	99

Table S4. Annotation of Peroxidase genes identified in the genome of *Pandoraea* sp. ISTKB

Pando_Id (query)Locus tag	NR_Db_Id (Subject)	Description	% Identity	alignment length	E- value	Query cover
A9762_15595	KGY27344.1	alkylhydroperoxidase [Achromobacterxylosoxidans]	89.308	159	1.15E-102	99
A9762_26215	SAK99084.1	alkylhydroperoxidase [Burkholderiapedi]	85.638	188	1.33E-112	99
A9762_04630	AMH44149.1	alkylhydroperoxidase [Burkholderia sp. PAMC 26561]	86.154	130	4.32E-82	99
A9762_16100	ODV41293.1	alkylhydroperoxidase [Cupriavidus sp. UYMMa02A]	77.835	194	4.87E-111	98
A9762_17195	SCB13161.1	alkylhydroperoxidaseAhpD family core domain-containing protein [Cupriavidusalkaliphilus]	70.909	110	1.67E-50	96
A9762_19885	AJA66520.1	chloroperoxidase [Bradyrhizobiumjaponicum]	73.934	211	3.59E-118	98
A9762_26475	AHB74970.1	glutathione peroxidase [Pandoraea pnomenusa]	90.446	157	1.7E-104	93
A9762_00985	AJG23567.1	Non-hemechloroperoxidase [Cupriavidusbasilensis]	81.55	271	2.31E-168	98
A9762_20355	EPX97329.1	peroxidase [Ralstonia sp. AU12-08]	87.736	212	1.07E-136	99
A9762_25345	EFF74736.1	peroxidase-like protein [Achromobacterpiechaudii ATCC 43553]	65.031	163	3.94E-66	91
A9762_24250	ALL66474.1	putative dye-decolorizing peroxidase (DyP), encapsulated subgroup [Paraburkholderiacaribensis MBA4]	70.492	366	0	96
A9762_25350	SCB32374.1	uncharacterized peroxidase-related enzyme [Cupriavidusalkaliphilus]	82.234	197	8.13E-119	99
A9762_16100	EIF34546.1	putativeperoxidase-relatedenzyme[Burkholderiasp.Ch1-1]	77.72	193	2.49E-110	99
A9762_16100	SAL70254.1	peroxidase-likeprotein[Paraburkholderiachoica]	76.684	193	1.17E-108	99
A9762_12720	KPD15963.1	thiolperoxidase[Burkholderiasp.ST111]	74.545	165	4.46E-84	99
A9762_19740	AKN73102.1	chloroperoxidase[Streptomycessp.PBH53]	57.075	212	2.87E-91	91
A9762_04630	AFT89771.1	AhpDfamilyalkylhydroperoxidase-likeprotein[ParaburkholderiaphenoliruptrixBR3459a]	86.923	130	1.74E-80	99

Table S5. Peripheral pathways for catabolism of aromatic compounds

Pando_Id (query)Locus tag	NR_Db_Id (Subject)	Description	% Identity	Alignment length	E-value	Query cover
**Salicylate ester degradation**						
A9762_00930	ODV40846.1	FAD-dependent oxidoreductase [Cupriavidus sp. UYMMa02A] , salicylate hydroxylase	77.601	567	0	98
A9762_05250	AOL06528.1	6-hydroxynicotinate 3-monooxygenase [Burkholderialata] , salicylate hydroxylase	84.555	382	0	98
A9762_27205	WP_044453514.1	monooxygenase [Pandoraea vervacti], salicylate hydroxylase	88	504	0	99
A9762_10935	ODS76726.1	salicylate hydroxylase [Bordetella sp. SCN 67-23]	75.346	361	0	94
**Toluene degradation**						
A9762_20020	CUV47131.1	toluenesulfonate zinc dependent alcohol dehydrogenase, [Ralstoniasolanacearum]	74.704	253	2.15E-134	99
**Quinate degradation**						
A9762_12745	EON15457.1	3-dehydroquinate dehydratase [Pandoraea sp. SD6-2]	95.27	148	2.28E-99	99
**Benzoate degradation**						
A9762_14900	SAL34481.1	major facilitator transporter [Paraburkholderiasordidicola]	81.551	477	0	99
A9762_00885	EKD98002.1	hypothetical protein ACD_23C00630G0002 [uncultured bacterium]	78.36	439	0	95
A9762_08025	EON13600.1	major facilitator transporter [Pandoraea sp. SD6-2]	93.432	472	0	99
A9762_02810	EON12244.1	benzoate transporter [Pandoraea sp. SD6-2]	96.615	384	0	96
A9762_21250	AHB76015.1	benzoylformate decarboxylase [Pandoraea pnomenusa]	89.114	542	0	99
A9762_16340	AMP38069.1	salicylate hydroxylase [Ralstoniasolanacearum]	64.375	160	7.55E-77	99
A9762_16345	KHQ96185.1	salicylate hydroxylase [Pandoraea pnomenusa]	94.393	428	0	99
**p-Hydroxybenzoate degradation**						
A9762_17475	SAL13664.1	4-hydroxybenzoate 3-monooxygenase [Paraburkholderiatelluris]	67.263	391	0	99
A9762_17480	AKQ44180.1	4-hydroxybenzoate transporter [Cupriavidus sp. ST-14]	68.224	428	0	93
A9762_19875	SAK47250.1	major facilitator transporter [Burkholderiaarationis], 4-hydrozybenzoate transporter	52.381	462	5.64E-161	99
A9762_01360	SCB18308.1	MFS transporter, AAHS family, 4-hydroxybenzoate transporter [Cupriavidusalkaliphilus]	76.906	446	0	99
A9762_01395	AJC15953.1	4- hydroxybenzoate transporter, hypothetical protein NA29_07520 [Pandoraea sputorum]	92.601	446	0	99
A9762_05365	AHB77010.1	MFS transporter [Pandoraea pnomenusa], 4- hydroxybenzoate transporter	90.95	442	0	97
A9762_06695	AJC22813.1	4-hydroxybenzoate transporter [Pandoraea pulmonicola]	95.122	451	0	99

Pando_Id (query)Locus tag	NR_Db_Id (Subject)	Description	% Identity	Alignment length	E-value	Query cover
**Gentisate degradation**						
A9762_01340	SCB18330.1	gentisate 1,2-dioxygenase [Cupriavidusalkaliphilus]	84.77	348	0	99
A9762_14650	CDO29336.1	2-hydroxyhepta-2,4-diene-1,7-dioate isomerase [Mycobacterium vulneris]	72.174	230	3.71E-119	98
A9762_19175	CFB64090.1	Ureidoglycolatelyase [Pandoraea apista]	89.274	317	0	99
A9762_23215	EHP44713.1	fumarylacetoacetate (FAA) hydrolase [Cupriavidusbasilensis OR16]	63.137	255	1.79E-117	99
A9762_27350	SAL20802.1	fumarylacetoacetate (FAA) hydrolase [Paraburkholderiatelluris]	82.927	328	0	99
A9762_01350	ODV43763.1	maleylacetoacetateisomerase [Cupriavidus sp. UYMMa02A]	74.648	213	1.87E-117	99
A9762_03985	AKM33115.2	Glutathione S-transferase, zeta (EC 2.5.1.18)	81.86	215	7.24E-126	99
A9762_02055	AHB76272.1	maleylacetoacetateisomerase [Pandoraea pnomenusa]	89.623	212	7.34E-142	98
A9762_01355	SCB18315.1	3-hydroxybenzoate 6-hydroxylase [Cupriavidusalkaliphilus]	83.733	375	0	92
A9762_17480	AKQ44180.1	4-hydroxybenzoate transporter [Cupriavidus sp. ST-14]	68.224	428	0	93
A9762_19875	SAK47250.1	4- hydroxybezoate transporter [Burkholderiaarationis]	52.381	462	5.64E-161	99
A9762_01360	SCB18308.1	4-hydroxybenzoate transporter [Cupriavidusalkaliphilus]	76.906	446	0	99
A9762_01395	AJC15953.1	4-hydroxybenzoate transporter	92.601	446	0	99
A9762_05365	AHB77010.1	MFS transporter [Pandoraea pnomenusa]	90.95	442	0	97
A9762_06695	WP_044456298.1	MFS transporter [Pandoraea vervacti]	98	440	0	100
**Chloroaromatic degradation pathway**						
A9762_14800	AHB74276.1	3-oxoadipate enol-lactone hydrolase [Pandoraea pnomenusa]	90.975	277	0	95
A9762_17465	AJP59451.1	3-oxoadipate enol-lactonase [Pandoraea vervacti]	91.373	255	1.08E-168	97
A9762_20400	AJC18736.1	hypothetical protein NA29_10045 [Pandoraea sputorum]	86.296	270	1.18E-155	94
A9762_21335	CFB62067.1	Putative aminoacrylate hydrolase RutD [Pandoraea apista]	85.833	240	6.85E-132	97
A9762_22015	ALS60105.1	hypothetical protein AT302_10360 [Pandoraea norimbergensis]	83.267	251	2.22E-142	95
A9762_00955	SCB31925.1	acetyl-CoA acyltransferase [Cupriavidusalkaliphilus]	85.75	400	0	99
A9762_23730	SCB31925.1	acetyl-CoA acyltransferase [Cupriavidusalkaliphilus]	86	400	0	99
A9762_00950	SCB31929.1	3-oxoadipate CoA-transferase beta subunit [Cupriavidusalkaliphilus]	84.211	209	2.42E-128	97
A9762_00945	SAL68494.1	3-oxoacid CoA-transferase subunit A [Paraburkholderiatelluris]	88.288	222	1.13E-140	98

Contd ……

Pando_Id (query)Locus tag	NR_Db_Id (Subject)	Description	% Identity	Alignment length	E-value	Query cover
**Aromatic Amin Catabolism**						
A9762_24855	ODS79332.1	3,4-dihydroxyphenylacetate 2,3-dioxygenase (EC 1.13.11.15)	72.222	288	2.28E-159	99
A9762_23735	WP_044457180.1	phenylacetic acid degradation protein PaaN [Pandoraea vervacti]	98	551	0	100
A9762_14815	AJP59439.1	flavinreductase/ nitrilotriacetatemonooxygenase [Pandoraea vervacti]	94.603	315	0	99
A9762_03435	SAL10420.1	Nitrilotriacetatemonooxygenase component B (EC 1.14.13.-)	80.976	205	2.7E-119	98
A9762_20390	AJC19509.1	flavinreductase [Pandoraea pulmonicola]	93.605	172	5.42E-118	96
A9762_05695	SAL83234.1	4-hydroxyphenylacetate 3-monooxygenase, reductase component (EC 1.6.8.-)	68.421	152	6.97E-72	88
A9762_05815	AMH43479.1	flavinreductase domain-containing protein [Burkholderia sp. PAMC 26561]	81.026	195	4.32E-114	92
A9762_00395	ERH61157.1	flavinreductase [Pseudomonas fluorescens EGD-AQ6]	84.337	166	4.13E-98	99
**Lignin degradation fragments**						
A9762_21325	EON13924.1	4-carboxy-4-hydroxy-2-oxoadipate aldolase (EC 4.1.3.17)	96.996	233	9.44E-161	99
A9762_21330	CDQ34089.1	4-oxalomesaconate tautomerase/ FldA protein [Virgibacillushalodenitrificans]	66.667	369	2.24E-164	99
A9762_21290	ALS59920.1	LysR family transcriptional regulator associated with utilization of aromatics[Pandoraea norimbergensis]	94.395	446	0	99
A9762_21285	ODS69252.1	Protocatechuate 4,5-dioxygenase alpha chain (EC 1.13.11.8)	71.429	119	5.36E-57	99
A9762_21280	EGD14970.1	protocatechuate 4,5-dioxygenase beta subunit [Xanthomonasperforans 91-118]	82.394	284	4.95E-175	99
**Vanillin degradation**						
A9762_11020	SCB20362.1	ferredoxin--NADP+ reductase/vanillate O-demethylaseferredoxin subunit [Cupriavidusalkaliphilus]	59.248	319	4.01E-128	99
A9762_21255	EON13938.1	Probable VANILLIN dehydrogenase oxidoreductase protein (EC 1.-.-.-)	93.047	489	0	99
A9762_21265	SCK14327.1	vanillate O-demethylaseferredoxin subunit [Variovorax sp. HW608]	63.208	318	7.8E-141	99
A9762_21270	CUV18877.1	vanillate O-demethylaseoxygenase subunit (4-hydroxy-3-methoxybenzoate demethylase) [Ralstoniasolanacearum]	74.194	341	0	96
A9762_21275	AHB76020.1	Transcriptional regulator for ferulate or vanillate catabolism	96.698	212	8.71E-144	95
**Ferulate degradation**						
A9762_19045	EON11379.1	feruloyl-CoA synthase [Pandoraea sp. SD6-2]	87.84	625	0	99
A9762_21275	AHB76020.1	Transcriptional regulator for ferulate or vanillate catabolism	96.698	212	8.71E-144	95

Pando_Id (query)Locus tag	NR_Db_Id (Subject)	Description	% Identity	Alignment length	E-value	Query cover
**Aromatic amino acid degradation**						
A9762_05715	KDR37901.1	pyridoxal-dependent decarboxylase [Paraburkholderiaglathei]	75.771	454	0	98
A9762_24850	ODS79333.1	5-carboxymethyl-2-hydroxymuconate semialdehyde dehydrogenase (EC 1.2.1.60)	76.375	491	0	99
A9762_24855	ODS79332.1	3,4-dihydroxyphenylacetate 2,3-dioxygenase (EC 1.13.11.15)	72.222	288	2.28E-159	99
A9762_24840	ODS79336.1	2-oxo-hepta-3-ene-1,7-dioic acid hydratase [Acidovorax sp. SCN 65-28]	71.429	266	1.65E-139	99
A9762_14090	ODT32327.1	2,4-dihydroxyhept-2-ene-1,7-dioic acid aldolase (EC 4.1.2.-)	39.357	249	3.06E-45	87
A9762_00725	SAL80058.1	Aromatic amino acid transport protein AroP	76.744	473	0	99
A9762_00975	SAK78922.1	aromatic amino acid transport protein [Burkholderiaptereochthonis]	88.641	449	0	97
A9762_04905	AJC18741.1	Phenylalanine-specific permease	71.336	464	0	97
A9762_22710	AJC18741.1	Phenylalanine-specific permease	97.216	467	0	99
A9762_20380	KHQ93930.1	kynurenineformamidase [Pandoraea pnomenusa]	92.079	202	2.78E-132	98
A9762_13280	EON12850.1	phenylalanine 4-monooxygenase [Pandoraea sp. SD6-2]	93.95	281	0	99
A9762_00970	ODV41070.1	4-hydroxyphenylpyruvate dioxygenase [Cupriavidus sp. UYMMa02A]	91.317	357	0	99
A9762_20370	AJP59879.1	tryptophan 2,3-dioxygenase [Pandoraea vervacti]	90.282	319	0	97
A9762_26260	ODV14183.1	2-hydroxyhepta-2,4-diene-1,7-dioate isomerase [Rubrivivax sp. SCN 70-15]	69.338	287	8.14E-141	99
A9762_13435	WP_039394138.1	homoprotocatechuate degradation operon regulator, HpaR [Pandoraea]	98	161	1.00E-112	100
A9762_01460	CAJ95344.1	2-Hydroxymuconic semialdehyde dehydrogenase [Ralstoniaeutropha H16]	85.744	484	0	99
**Benzoyl-CoA mediated degradation**						
A9762_07750	KPD16106.1	benzoyl-CoA oxygenase [Burkholderia sp. ST111]	92.421	475	0	99
A9762_07755	AFT86541.1	benzoyl-CoA 2,3-dioxygenase component A [Paraburkholderiaphenoliruptrix BR3459a]	75.708	424	0	99
A9762_12180	CRM37160.1	anaerobic benzoate catabolism transcriptional regulator [Pseudomonas sp. 58 R 3]	38.356	73	8.07E-11	88
A9762_08410	KHQ95703.1	4-hydroxybenzoyl-CoA thioesterase [Pandoraea pnomenusa]	97.183	142	4.08E-98	90
A9762_19020	EON11384.1	anaerobic benzoate catabolism transcriptional regulator [Pandoraea sp. SD6-2]	91.815	281	0	99
A9762_23595	CRM37160.1	anaerobic benzoate catabolism transcriptional regulator [Pseudomonas sp. 58 R 3]	33.721	86	2.23E-08	90
A9762_08410	KHQ95703.1	4-hydroxybenzoyl-CoA thioesterase [Pandoraea pnomenusa]	97.183	142	4.08E-98	90

Contd….

Pando_Id (query)Locus tag	NR_Db_Id (Subject)	Description	% Identity	Alignment length	E-value	Query cover
**Biphenyl or cinnamic acid degradation**						
A9762_10790	AJC19190.1	2-keto-4-pentenoate hydratase [Pandoraea sputorum]	97.77	269	0	95
A9762_19185	AHB78072.1	2,3-dihydroxybiphenyl 1,2-dioxygenase	92.973	185	3.29E-127	99
A9762_04960	ODS74842.1	4-hydroxy-2-oxovalerate aldolase [Bordetella sp. SCN 67-23]	89.254	335	0	99
A9762_25270	AJP58375.1	2-hydroxy-6-oxo-6-phenylhexa-2,4-dienoate hydrolase (EC 3.7.1.-)	86.88	343	0	97
A9762_25765	AHB77129.1	2-keto-4-pentenoate hydratase (EC 4.2.1.80)	80	220	2.77E-124	87
**Phenol degradation**						
A9762_15430	ODS71808.1	2-polyprenyl-6-methoxyphenol hydroxylase and related FAD-dependent oxidoreductases	62.532	387	2.28E-161	94
A9762_20610	BAK84768.1	2-polyprenyl-6-methoxyphenol hydroxylase and related FAD-dependent oxidoreductases	57.538	398	4.94E-162	99
A9762_03525	WP_046291377.1	Putative MetA-pathway of phenol degradation (pfam)	93	231	2.00E-169	100
A9762_27630	WP_047905605.1	Putative MetA-pathway of phenol degradation (pfam)	90	218	1.00E-161	100
**Phenylacetic acid degradation**						
A9762_14315	SCB11103.1	phenylacetate-CoA ligase [Cupriavidusalkaliphilus]	65.468	417	0	99
A9762_23715	SAK91506.1	phenylacetate-CoA ligase [Burkholderiaarationis]	85.581	430	0	99
A9762_23720	EON10727.1	phenylacetic acid degradation- related thioesterase (PaaI) [Pandoraea sp. SD6-2]	93.525	139	7.92E-94	99
A9762_23865	GAT99760.1	phenylacetate-CoA oxygenase subunit PaaB [Burkholderiastabilis]	86.022	93	1.01E-54	94
A9762_23875	CFB61605.1	Putative 1,2-phenylacetyl-CoA epoxidase, subunit D [Pandoraea apista]	96.316	190	8.62E-132	98
A9762_23880	SAL63160.1	phenylacetate-CoA oxygenase/reductase subunit PaaK [Paraburkholderiatelluris]	72.099	362	0	99
A9762_27195	AJC15727.1	4-hydroxyphenylacetate permease [Pandoraea sputorum]	94.651	430	0	99
**Phenylpropionic acid and cinnamic acid degradation**						
A9762_11010	ALU88520.1	Phenylpropionatedioxygenase,largeterminalsubunit[Variovoraxsp.HW608]	77.108	332	0	97

Table S6. Annotation of genes responsible for metabolism of central aromatic intermediates

Catechol branch of beta-ketoadipate pathway

Pando_Id (query)Locus tag	NR_Db_Id (Subject)	Description	% Identity	Alignment length	E-value	Query cover
A9762_19835	EPX97674.1	Catechol 1,2-dioxygenase (EC 1.13.11.1)	79.273	275	4.23E-160	94
A9762_05645	BAM71411.1	hydroxyquinol 1,2-dioxygenase [Ralstoniapickettii]	72.263	274	4.7E-144	94
A9762_13825	KAK75849.1	mandelateracemase/muconatelactonizing enzyme [Bordetellabronchiseptica MO211]	84.677	372	0	99
A9762_14800	AHB74276.1	3-oxoadipate enol-lactone hydrolase [Pandoraeapnomenusa]	90.975	277	0	95
A9762_17465	AJP59451.1	3-oxoadipate enol-lactonase [Pandoraeavervacti]	91.373	255	1.08E-168	97
A9762_20400	AJC18736.1	Beta-ketoadipateenol-lactone hydrolase (EC 3.1.1.24)	86.296	270	1.18E-155	94
A9762_21335	CFB62067.1	Putative aminoacrylate hydrolase RutD [Pandoraeaapista]	85.833	240	6.85E-132	97
A9762_00945	SAL68494.1	3-oxoacid CoA-transferase subunit A [Paraburkholderiatelluris]	88.288	222	1.13E-140	98
A9762_26655	SCB24496.1	3-oxoacid CoA-transferase subunit A [Cupriavidusalkaliphilus]	87.446	231	1.69E-147	99
A9762_22015	ALS60105.1	Succinyl-CoA:3-ketoacid-coenzyme A transferase subunit A (EC 2.8.3.5)	83.267	251	2.22E-142	95
A9762_00950	SCB31929.1	3-oxoadipate CoA-transferase beta subunit [Cupriavidusalkaliphilus]	84.211	209	2.42E-128	97
A9762_26660	SAL73718.1	3-oxoacid CoA-transferase subunit B [Paraburkholderiachoica]	89.151	212	1.94E-133	99

Salicylate and gentisate catabolism

Pando_Id (query)Locus tag	NR_Db_Id (Subject)	Description	% Identity	Alignment length	E-value	Query cover
A9762_00930	ODV40846.1	FAD-dependent oxidoreductase [Cupriavidus sp. UYMMa02A]	77.601	567	0	98
A9762_01340	SCB18330.1	gentisate 1,2-dioxygenase [Cupriavidusalkaliphilus]	84.77	348	0	99
A9762_01355	SCB18315.1	3-hydroxybenzoate 6-hydroxylase [Cupriavidusalkaliphilus]	83.733	375	0	92
A9762_05250	AOL06528.1	6-hydroxynicotinate 3-monooxygenase [Burkholderialata]	84.555	382	0	98
A9762_27205	WP_044453514.1	monooxygenase [Pandoraeavervacti]	88	504	0	99
A9762_10935	ODS76726.1	salicylate hydroxylase [Bordetella sp. SCN 67-23]	75.346	361	0	94
A9762_17480	AKQ44180.1	4-hydroxybenzoate transporter [Cupriavidus sp. ST-14]	68.224	428	0	93
A9762_19875	SAK47250.1	major facilitator transporter [Burkholderiaarationis]	52.381	462	5.64E-161	99
A9762_01360	SCB18308.1	4-hydroxybenzoate transporter [Cupriavidusalkaliphilus]	76.906	446	0	99
A9762_01395	AJC15953.1	4-hydroxybenzoate transporter	92.601	446	0	99
A9762_05365	AHB77010.1	MFS transporter [Pandoraeapnomenusa]	90.95	442	0	97
A9762_06695	AJC22813.1	4-hydroxybenzoate transporter [Pandoraeapulmonicola]	95.122	451	0	99
A9762_01350	ODV43763.1	maleylacetoacetateisomerase [Cupriavidus sp. UYMMa02A]	74.648	213	1.87E-117	99
A9762_03985	AKM33115.2	Maleylacetoacetateisomerase (EC 5.2.1.2)	81.86	215	7.24E-126	99
A9762_05255	WP_039397187.1	Asp/Gluracemase [Pandoraea_sputorum]	97	243	5.00E-176	100
A9762_02055	AHB76272.1	maleylacetoacetateisomerase [Pandoraeapnomenusa]	89.623	212	7.34E-142	98
A9762_01345	ODV43764.1	5-carboxymethyl-2-hydroxymuconate isomerase [Cupriavidus sp.	84.483	232	1.8E-144	99
A9762_21820	SCB17386.1	fumarylacetoacetate hydrolase [Cupriavidusalkaliphilus]	73.934	422	0	99
A9762_14650	CDO29336.1	2-hydroxyhepta-2,4-diene-1,7-dioate isomerase [Mycobacterium vulneris]	72.174	230	3.71E-119	98
A9762_19175	CFB64090.1	Ureidoglycolatelyase [Pandoraeaapista]	89.274	317	0	99
A9762_23215	EHP44713.1	fumarylacetoacetate (FAA) hydrolase [Cupriavidusbasilensis OR16]	63.137	255	1.79E-117	99
A9762_27350	SAL20802.1	fumarylacetoacetate (FAA) hydrolase [Paraburkholderiatelluris]	82.927	328	0	99

Protocatechuate branch of beta-ketoadipate pathway

Pando_Id (query)Locus tag	NR_Db_Id (Subject)	Description	% Identity	Alignment length	E-value	Query cover
A9762_00940	AJC18662.1	IclR family transcriptional regulator [Pandoraeasputorum]	97.276	257	8.39E-180	94
A9762_17445	OED18908.1	protocatechuate 3,4-dioxygenase subunit beta [Burkholderia sp. A2]	74.569	232	2.71E-126	98
A9762_17450	WP_046291001.1	protocatechuate 3,4-dioxygenase subunit alpha [Pandoraeaoxalativorans]	89	178	2.00E-130	100
A9762_17455	AHB76694.1	3-carboxy-cis,cis-muconate cycloisomerase [Pandoraeapnomenusa]	84.464	457	0	99
A9762_14775	ODT36810.1	4-carboxymuconolactone decarboxylase [Lautropia sp. SCN 70-15]	80.833	120	1.42E-68	96
A9762_17460	CAQ89147.1	4-carboxymuconolactone decarboxylase (CMD) [Escherichia fergusonii ATCC 35469]	68.75	128	2.55E-61	88
A9762_14800	AHB74276.1	3-oxoadipate enol-lactone hydrolase [Pandoraeapnomenusa]	90.975	277	0	95
A9762_17465	AJP59451.1	3-oxoadipate enol-lactonase [Pandoraeavervacti]	91.373	255	1.08E-168	97
A9762_20400	AJC18736.1	Beta-ketoadipateenol-lactone hydrolase (EC 3.1.1.24)	86.296	270	1.18E-155	94
A9762_21335	CFB62067.1	Putative aminoacrylate hydrolase RutD [Pandoraeaapista]	85.833	240	6.85E-132	97
A9762_22015	ALS60105.1	Beta-ketoadipateenol-lactone hydrolase (EC 3.1.1.24)	83.267	251	2.22E-142	95
A9762_00945	SAL68494.1	3-oxoacid CoA-transferase subunit A [Paraburkholderiatelluris]	88.288	222	1.13E-140	98
A9762_00950	SCB31929.1	3-oxoadipate CoA-transferase beta subunit [Cupriavidusalkaliphilus]	84.211	209	2.42E-128	97
A9762_00955	SCB31925.1	acetyl-CoA C-acetyltransferase/acetyl-CoA acyltransferase [Cupriavidusalkaliphilus]	85.75	400	0	99
A9762_23730	SCB31925.1	acetyl-CoA C-acetyltransferase/acetyl-CoA acyltransferase [Cupriavidusalkaliphilus]	86	400	0	99
A9762_26655	SCB24496.1	3-oxoacid CoA-transferase subunit A [Cupriavidusalkaliphilus]	87.446	231	1.69E-147	99
A9762_26660	SAL73718.1	3-oxoacid CoA-transferase subunit B [Paraburkholderiachoica]	89.151	212	1.94E-133	99
A9762_01305	AJC23270.1	alpha-ketoglutaratepermease [Pandoraeapulmonicola]	93.24	429	0	98
A9762_10400	AJC18570.1	MFS transporter / dicarboxylic acid transporter [Pandoraeasputorum]	97.375	419	0	99

N-heterocyclic aromatic compound degradation

Pando_Id (query)Locus tag	NR_Db_Id (Subject)	Description	% Identity	Alignment length	E-value	Query cover
A9762_12915	CFB61120.1	Isoquinoline 1-oxidoreductase subunit beta [Pandoraeaapista]	87.834	748	0	99
A9762_05275	WP_046292552.1	aldehyde dehydrogenase [Pandoraeaoxalativorans]	92	698	0	98
A9762_00300	AHB76506.1	Isoquinoline 1-oxidoreductase beta subunit (EC 1.3.99.16)	90.43	721	0	97
A9762_12910	AEF88978.1	Isoquinoline 1-oxidoreductase [Delftia sp. Cs1-4]	73.469	147	3.13E-75	99
A9762_05245	KPD19386.1	(2Fe-2S)-binding protein [Burkholderia sp. ST111]	80.488	164	6.22E-91	98
A9762_00295	WP_047908730.1	PLP-dependent aminotransferase family protein [Pandoraeafaecigallinarum]	94	376	0	100

Central meta-cleavage pathway of aromatic compound degradation

Pando_Id (query)Locus tag	NR_Db_Id (Subject)	Description	% Identity	Alignment length	E-value	Query cover
A9762_21285	ODS69252.1	Protocatechuate 4,5-dioxygenase alpha chain (EC 1.13.11.8)	71.429	119	5.36E-57	99
A9762_21280	EGD14970.1	protocatechuate 4,5-dioxygenase beta subunit [Xanthomonasperforans 91-118]	82.394	284	4.95E-175	99
A9762_24855	ODS79332.1	3,4-dihydroxyphenylacetate 2,3-dioxygenase (EC 1.13.11.15)	72.222	288	2.28E-159	99
A9762_24850	ODS79333.1	5-carboxymethyl-2-hydroxymuconate semialdehyde dehydrogenase (EC 1.2.1.60)	76.375	491	0	99
A9762_25270	AJP58375.1	2-hydroxy-6-oxo-6-phenylhexa-2,4-dienoate hydrolase (EC 3.7.1.-)	86.88	363	0	97
A9762_04685	WP_046292026.1	4-oxalocrotonate tautomerase [Pandoraeaoxalativorans]	93	65	2.00E-39	98
A9762_24840	ODS79336.1	2-oxo-hepta-3-ene-1,7-dioic acid hydratase [Acidovorax sp. SCN 65-28]	71.429	266	1.65E-139	99
A9762_04965	CFB59979.1	Acetaldehyde dehydrogenase [Pandoraeaapista]	98.408	314	0	99

Homogentisate pathway of aromatic compound degradation

Pando_Id (query)Locus tag	NR_Db_Id (Subject)	Description	% Identity	Alignment length	E-value	Query cover
A9762_21815	SAL80458.1	homogentisate 1,2-dioxygenase [Paraburkholderiatelluris]	82.944	428	0	97
A9762_00970	ODV41070.1	4-hydroxyphenylpyruvate dioxygenase [Cupriavidus sp. UYMMa02A]	91.317	357	0	99
A9762_01350	ODV43763.1	maleylacetoacetateisomerase [Cupriavidus sp. UYMMa02A]	74.648	213	1.87E-117	99
A9762_01310	KGW48977.1	merR regulatory family protein [Burkholderiapseudomallei MSHR684]	56.418	335	1.31E-127	97
A9762_02055	AHB76272.1	maleylacetoacetateisomerase [Pandoraeapnomenusa]	89.623	212	7.34E-142	98
A9762_01345	ODV43764.1	5-carboxymethyl-2-hydroxymuconate isomerase [Cupriavidus sp. UYMMa02A]	84.483	232	1.8E-144	99
A9762_21820	SCB17386.1	fumarylacetoacetate hydrolase [Cupriavidusalkaliphilus]	73.934	422	0	99
A9762_02750	SAL81482.1	aromatic amino acid aminotransferase [Paraburkholderiachoica]	78.697	399	0	99
A9762_02865	SAL18442.1	class I/II aminotransferase [Burkholderiaperedens]	69.531	384	0	97
A9762_00980	SAK78909.1	aromatic amino acid aminotransferase [Burkholderiaptereochthonis]	81.108	397	0	99
A9762_17555	SAL18442.1	class I/II aminotransferase [Burkholderiaperedens]	56.186	388	2.2E-160	95
A9762_01455	ODV41069.1	aromatic amino acid aminotransferase [Cupriavidus sp. UYMMa02A]	74.747	396	0	99
A9762_24875	SAL50293.1	IclR family transcriptional regulator [Paraburkholderiaudeis]	80.992	242	3.32E-140	96
A9762_14885	CFB62532.1	Acetate operon repressor [Pandoraeaapista]	97.71	262	0	99
A9762_00805	SAL80324.1	IclR family transcriptional regulator [Paraburkholderiatelluris]	68.539	267	1.59E-125	92
A9762_00810	SCB09651.1	transcriptional regulator, IclR family [Cupriavidusalkaliphilus]	70.238	252	3.65E-122	99
A9762_03330	AHB76430.1	IclR family transcriptional regulator [Pandoraeapnomenusa]	85.502	269	7.89E-152	95
A9762_00915	SAL87254.1	IclR family transcriptional regulator [Paraburkholderiachoica]	81.102	254	1.05E-150	99
A9762_17580	AKM32377.2	Transcriptional regulator, IclR family	98.77	244	4.71E-174	99
A9762_19060	AHB76961.1	transcriptional regulator [Pandoraeapnomenusa]	90.476	252	4.31E-167	86
A9762_19985	AHB74593.1	IclR family transcriptional regulator [Pandoraeapnomenusa]	95.455	264	0	99
A9762_20365	CFB60313.1	HTH-type transcriptional regulator KipR [Pandoraeaapista]	98.79	248	2.94E-178	99
A9762_20415	AHB74544.1	IclR family transcriptional regulator [Pandoraeapnomenusa]	98.387	248	9.38E-179	99
A9762_22920	EON11650.1	IclR family transcriptional regulator [Pandoraea sp. SD6-2]	97.942	243	2.96E-174	99
A9762_23015	ALL68065.1	transcriptional regulator, IclR family [Paraburkholderiacaribensis MBA4]	63.035	257	1.41E-108	95
A9762_23460	OAK61863.1	IclR family transcriptional regulator [Variovoraxparadoxus]	43.154	241	1.14E-65	89
A9762_05760	KPD16152.1	IclR family transcriptional regulator [Burkholderia sp. ST111]	83.86	285	3.26E-170	96
A9762_25085	EON13327.1	IclR family transcriptional regulator [Pandoraea sp. SD6-2]	93.309	269	1.87E-177	99
A9762_06720	WP_039395317.1	IclR family transcriptional regulator [Pandoraeasputorum]	99	258	0	100
A9762_02330	WP_047906735.1	IclR family transcriptional regulator [Pandoraeafaecigallinarum]	89	269	0	100
A9762_27355	CFB62943.1	Transcriptional regulator, IclR family	85.56	277	3.57E-152	94
A9762_10930	AJP59478.1	IclR family transcriptional regulator [Pandoraeavervacti]	94.792	288	0	99
A9762_10970	AJP59478.1	IclR family transcriptional regulator [Pandoraeavervacti]	94.792	288	0	99

Table S7. Annotation of genes related to Glutathione metabolism and stress response

Pando_Id (query)	NR_Db_Id (Subject)	Description	% identity	alignment length	evalue	qcovs
A9762_23385	OED18901.1	glutathione ABC transporter permeaseGsiD [Burkholderia sp. A2]	88.298	282	0	99
A9762_17400	AHB76675.1	glutathione ABC transporter permeaseGsiD [Pandoraeapnomenusa]	95.035	282	0	98
A9762_26475	AHB74970.1	glutathione peroxidase [Pandoraeapnomenusa]	90.446	157	1.7E-104	93
A9762_15125	OED18129.1	glutathione S-transferase [Burkholderia sp. A2]	71.429	203	1.57E-103	94
A9762_25920	OED12452.1	glutathione S-transferase [Burkholderia sp. A2]	68.095	210	2.7E-101	97
A9762_18915	KPD19945.1	glutathione S-transferase [Burkholderia sp. ST111]	77.13	223	1.8E-125	99
A9762_25290	ODV42245.1	glutathione S-transferase [Cupriavidus sp. UYMMa02A]	63.362	232	5.47E-103	97
A9762_17280	AJC18614.1	glutathione S-transferase [Pandoraeasputorum]	86.916	214	2.72E-125	95
A9762_09395	SAL44491.1	glutathione S-transferase [Paraburkholderiatelluris]	55.399	213	2.37E-84	97
A9762_21360	KLN47139.1	glutathione S-transferase [Providenciarettgeri]	35.644	202	2.3E-39	92
A9762_00695	AIL61297.1	glutathione S-transferase [Pseudomonas alkylphenolia]	62.201	209	3.06E-86	99
A9762_01420	EJL96406.1	glutathione S-transferase [Pseudomonas sp. GM102]	61.194	201	1.33E-83	90
A9762_25435	OAK61673.1	glutathione S-transferase [Variovoraxparadoxus]	58.824	204	1.73E-79	99
A9762_27060	AIO26625.1	glutathione S-transferase 2 [Burkholderiacepacia ATCC 25416]	74.528	212	2.24E-115	92
A9762_02245	SAL21216.1	glutathione synthetase [Paraburkholderiatelluris]	75	316	4.09E-169	99
A9762_17125	KUK27111.1	Glutathione transport system permease protein GsiC [Acetothermia bacterium 64_32]	50.336	298	1.08E-96	95
A9762_17115	CDZ54459.1	Glutathione-binding protein GsiB [Neorhizobiumgalegaebv. orientalis]	56.89	508	0	95
A9762_15295	EPJ93917.1	glutathione-dependent formaldehyde-activating GFA [Pseudomonas sp. CF149]	56.154	130	6.78E-49	98
A9762_14280	ALK32265.1	glutathione-dependent formaldehyde-activating protein [Burkholderiaplantarii]	48.507	134	2.82E-44	98
A9762_06655	EIM72377.1	glutathione-dependent formaldehyde-activating protein [Nitratireductoraquibiodomus RA22]	55.856	111	1.05E-40	93
A9762_24585	ANY77517.1	glutathione-disulfidereductase [Microvirga sp. V5/3M]	64.143	449	0	99
A9762_19965	CEJ12736.1	Glutathione-regulated potassium-efflux system ancillary protein KefF [bacterium YEK0313]	65.5	200	2.42E-91	98
A9762_08325	AHB05231.2	hydroxyacylglutathione hydrolase [Pandoraeapnomenusa 3kgm]	88.462	260	9.54E-167	94
A9762_04000	KPD20186.1	lactoylglutathionelyase [Burkholderia sp. ST111]	70.161	124	3.06E-58	99
A9762_12990	CFB61104.1	Lactoylglutathionelyase [Pandoraeaapista]	93.985	133	1.45E-89	99

Superoxide Dismutase

Pando_Id (query)	NR_Db_Id (Subject)	Description	% identity	alignment length	evalue	qcovs
A9762_16420	KYP12368.1	superoxide dismutase [Limnobacter sp. CACIAM 66H1]	83.246	191	1.23E-120	99
A9762_20590	ODT64140.1	superoxide dismutase [Nitrosomonadales bacterium SCN 54-20]	55.959	193	3.81E-71	97
A9762_05205	WP_038617481.1	Superoxide dismutase SodM-like protein ChrF	88	218	5.00E-19	100

Glutaredoxin

Pando_Id (query)	NR_Db_Id (Subject)	Description	% identity	alignment length	evalue	qcovs
A9762_21380	SAK82439.1	Glutaredoxin [Burkholderiacatudaia]	38.672	256	4.31E-54	99
A9762_18155	SAL29912.1	glutaredoxin 2 [Burkholderiaperedens]	56.579	76	1.35E-22	93
A9762_02205	SAL83115.1	glutaredoxin 3 [Paraburkholderiachoica]	80.233	86	1.27E-46	99

Thioredoxin

Pando_Id (query)	NR_Db_Id (Subject)	Description	% identity	alignment length	evalue	qcovs
A9762_10565	EON13999.1	Putative thioredoxin [Pandoraea sp. SD6-2]	91.406	128	8.87E-79	98
A9762_02295	SAL26452.1	Thiol-disulfideisomerase/thioredoxin-like protein [Burkholderiaperedens]	70.414	169	3.46E-83	96
A9762_09775	GAQ30519.1	thioredoxin [Ralstonia sp. NT80]	89.815	108	2.08E-66	99
A9762_21390	SAK58934.1	thioredoxinreductase [Burkholderiaptereochthonis]	72.24	317	6.13E-174	99
A9762_14295	OED12953.1	thioredoxinreductase [Burkholderia sp. A2]	69.435	301	2.32E-147	99
A9762_19935	ABO53901.1	thioredoxinreductase [Burkholderiavietnamiensis G4]	82.595	316	0	99
A9762_21500	CFB64917.1	Thioredoxin-1 [Pandoraeaapista]	90.106	283	0	99
A9762_12750	CAJ94244.1	Thioredoxin-like protein [Ralstoniaeutropha H16]	53.125	160	3.11E-56	91

Catalase

Pando_Id (query)	NR_Db_Id (Subject)	Description	% identity	alignment length	evalue	qcovs
A9762_14510	KPD18059.1	catalase [Burkholderia sp. ST111]	66.022	362	7.98E-175	99

A9762_15065	SCB07688.1	catalase [Cupriavidusalkaliphilus]	76.744	473	0	98
A9762_01165	AKB40108.1	Catalase [Methanosarcinamazei S-6]	59.468	301	1.76E-127	89
A9762_05565	ADE10952.1	Catalase [Sideroxydanslithotrophicus ES-1]	56.452	310	1.24E-126	86
A9762_19890	KUO98787.1	catalase [Stenotrophomonasmaltophilia]	92.139	547	0	99
A9762_17205	ODU43538.1	catalase [Thiobacillus sp. SCN 63-374]	84.034	476	0	97

Table S8. Represents secondary metabolite clusters identified in *Pandoraea* sp. ISTKB genome.

Clusters	Secondary metabolites	Contig number	Position	
			Start	End
Cluster 1	Terpene	NZ_MAOS01000023.1	43156	64031
Cluster 2	Nonribosomal peptides (Nrps)	NZ_MAOS01000028.1	266130	354115
Cluster 3	Thailanstatin/ Mangotoxin	NZ_MAOS01000031.1	42017	85616
Cluster 4	Arylpolyene	NZ_MAOS01000044.1	14082	59545
Cluster 5	Homoserine lactone	NZ_MAOS01000045.1	39255	60139
Cluster 6	Phosphonate-Terpene	NZ_MAOS01000050.1	6780	70269
Cluster 7	Bacteriocin	NZ_MAOS01000052.1	8006	18836
Cluster 8	Homoserine lactone	NZ_MAOS01000061.1	19488	40315
Cluster 9	Lassopeptide	NZ_MAOS01000083.1	160463	183061
